# Covered *versus* uncovered endoluminal stenting in the acute management of obstructing colorectal cancer in the palliative setting: randomized clinical trial (CReST2)

**DOI:** 10.1093/bjs/znaf117

**Published:** 2025-09-17

**Authors:** CReST2 Collaborative Group, James Hill, Nicola Fearnhead, Richard Gray, Kelly Handley, Manjinder Kaur, Clive Kay, Hans-Ulrich Laasch, Andrew Lowe, Laura Magill, Dion Morton, Ruben Mujica-Mota, Andy Palmer, Anne Pullyblank, Yongzhong Sun, Suresh Vasan Venkatachalapathy, Pete Wheatstone, Yasmin Ali, Altus Chan, Suzanne Locker, Paul Riley, Gordon Carlson, Stuart Taylor, Sarah Barry, Philip Bell, Steve Halligan, Nigel Scott, Suhail Ahmed, James Arthur, Carol Brooks, Jane L Hughes, Tanya Ingram, Michelle Linforth, Sophie Marsh, Rizwan Saleem, Simone Slawik, Sarah Stevenson, Lilian Wajero, Nicholas Cross, Amanda Dell, Mandy Edwards, Angela Hall, Helen Hamilton, Nancy Hawkins, Heidi Lawson, Mark Robinson, Michelle Tayler, Rebecca Wallace, Sarah Wheatman, Joanna Wilson, Lindianne Aitken, Rhodri Codd, Joseph Hamill, Nancy Hawkins, Georgia Mallison, Steve McKain, Heeam Nassa, Claire Louise Price, Mark Robinson, Brian Stephenson, Keshav Swarnkar, Claire Triscott, Elaine Wall, Rebecca Wallace, Gethin Williams, Cerian Williams, Rommel Butawan, Joseph Huang, Sam King, Tina Mills-Baldock, Purushothaman Premchand, Alison Ray, Amy Barnett, Alexander Blackmore, Oliver Brennan, Melaine Caswell, Greta Van Doyvenvoorde, James Glen, Shamina Hankinson, Mark Hendrickse, Peter Isaacs, Ilianna Mamali, Senthil Murugesan, Marina Oprea, Chris Pemberton, Ella Riedel, Arunan Sivapataham, Wei Fen Tay, Lauren Thornborough, Rachel Wheeldon, Conor Wilkinson, Julie Chadwick, Shirley Cocks, Gemma Faulkner, Robert Hull, Marta Martinez Iglesias, James Lay, Ha Phuong Do Le, James Pollard, Shenraga Kumar Rajamanickam, Rubeena Razzaq, Michaela Sutherland, Aphan Abdulholim, Conrad Beckett, Wendy Cardozo, Carol Firth, Naeem Jagirdar, Wendy Jepson, Sarah Jowett, Amjad Mohammed, Sulleman Moreea, Nicolas Rabb, Jonathan Robinson, Sophie Stephenson, Sarah Tinker, Ashlea Bucke, Ewen Cameron, Nicholas Carroll, Gareth Corbett, Nicola Fearnhead, Nigel Hall, Alisa Liddle, Ines Modolell, Jonathan Morton, Aileen Nacorda, Sophie Newton, Beverley Nobbs, Debbie Read, Rebekka Troller, James Wheeler, Lucy Worboys, Megan Brickhill, Chris Craig, Finlay Curran, David Donnelly, Mona Fareh, Bethanie Garside, Glaxy Gray, Richard Hammonds, Babra Hanif, James Hill, Benjamin Hornung, Anu John, Maya John, Stephen Lee, Jesha Mathews, Pavenjit Nandhra, Mohammed Nazir, Jessica Nichols, Sarah O'Shea, Alice Panes, Laura Perry, Angelique Quistin, Rojy Santosh, Nicholas Stylianides, Jennifer Trezise, Denielle Wilcock, Gian Abbott, Paul Evans, Claire Gabriel, Jenny Grounds, Nichola Kearsley, Roy Mahapatra, Collette Markzu, Emmeline Martin, Laura Parry, Sandra Powell, Kunal Rajput, Dale Vimalachandran, Andrea Young, Helen Boros, Lisa Hardstaff, Philippa Hill, Maureen Holland, Debra Jowle, Konrad Koss, Barbara Townley, Lesley Wilknson, Hayley Cousins, Barbara King, John Ramage, Amanda Alty, Paul Barrow, Alan Beveridge, Arnab Bhowmick, Alistair Craig, Terri-Louise Cromie, Tarek Hany, Alka Jadav, Janet Mills, Peter Mitchell, Ed Parkin, Ioannis Peristerakis, Sandra Sowden, Robert Stockwell, Gagandeep Thind, Louis Turrel, Mark Verlander, Ailsa Watt, Deborah Weavers, Alexandra Williams, Miranda Baum, Simon Everett, Vinod Hegade, Matthew Huggett, Susan Kelly, Rebecca King, Lucy Marshall, Catherine Moriarty, Bharat Paranandi, Mark Priestley, Rick Saunders, Holly Speight, Louise White, Haidar Alwan-Walker, Helen Ashby, Linda Bailey, Wal Baraza, Molly Bennett, Angela Chrisopoulou, Sarah Duff, Laura Hancock, Zoe Holliday, Javaid Iqbal, Venkata Lekharaju, Fiona McCartin, Gorei Mccavil, Stephen Metcalfe, Heena Mistry, Lindsay Piper, Aswatha Ramesh, Velauthan Rudralingam, Abhiram Sharma, Kathryn Slevin, Karen Telford, Debbie West, Kate Whitehead, Peter Coyne, James Graham, Stephanie Grieveson, Ben Griffiths, Lorna Ingoe, Sam McDonald, Victoria Murtha, Adam Scadeng, Julia Scott, Elaine Stephenson, Vithusa Varnakillasingam, Nelson Wong, Avril Donaldson, Kathleen Macleod, Andrew Macleod, Joanna Matheson, Raymond Oliphant, Alastair Todd, Michael Walker, Angus Watson, Angie Balfour, Domenyk Brown, John Brush, Stephen Glancy, Sarah Goodbrand, Marion MacRury, Chinnappa Reddy, Doug Speake, Geoff Wogan, Sarah Bevins, Kirstie Bradburn, Caroline Burt, Neil Collin, Graham Collin, Laura Fox, Robert Healey, Mitchell Hopes, Shinu Jackson, Alice Jarvie, Regina Kageni, Suriya Kirkpatrick, Sam Loud, Eric Loveday, Ann Lyons, Kathryn McCarthy, Tom McGirr, Angus McNair, Peter Mezes, Emily Perry, Anne Pullyblank, Sosamma Robin, Maricruz Santamaria, Alice Smith, Andrew Smith, Louise Solomon, Haytham Sumrien, Isileli Tonga, Katherine Way, David Westwood, Guruprasad P Aithal, Andrew Baxter, Suzanne Henry, Martin James, Amardeep Khanna, Jodie Newham, Sian Kelly Parkes, Stephen Ryder, Ioannis Varmpompitis, Suresh Vasan Venkatachalapathy, Samantha Warburton, Sarah Askew, Kerry Atkinson, Madalina Chifu, Candy Coombe, Sophia Eloi, Clare Ferris, Elizabeth Firth, Caroline Goddard, Anne Griffiths, John Hancock, Angela Irving, Kirsty Maclean, John Madine, Corrine Penhaligon, Catherine Pentescost, Kirsty Prout, Rebecca Rogers, Rebecca Sargent, Anita Steele, Felicity Verma, Michael Agyemang, Sarah Bird, Steven Brown, Holly Caborn, Joyce Fofie, James Hampton, Faith Kibutu, Fred Lee, Cecilia Mason, Angeline Mbuyisa, Helen Newell, Viktoria Cripps, Thomas Edwards, Nicky Forsyth, Louise Hunt, Andrew Lowe, Paul Mackey, Rudi Matull, Alison Moss, Corinne Pawley, Tamlyn Russell, Maria Salter, Charmaine Shovelton, Edward Smyth, Angela Berry, Nicola Broome, Grant Caddy, John Eccles, Jennifer Foreman, Tony Tham, Alex Usher-Rea, Andrew Wray, Gail Young, Harry Bond, Theresa Taylor Emberton, Hans-Ulrich Laasch, Sue Lane, Damian Mullan, Lyne Robertson, Maria RoyoGamara, Marie Green, Brian McKaig, Shyam Menon, Rajinder Nayyar, Julie Roberts, Helen Steed, Andrew Veitch, Sarah Addison, Shazad Ashraf, Simon Bach, Anil Bagul, Elizabeth Bailey, Andrew Beggs, Colm Forde, Sharon Garner, Manijeh Ghods, Andrew McDarby, Claire McNeill, Dion Morton, Dimitri Nepogodiev, Arvind Pallan, Tom Pinkney, Jonathan Richardson, Nigel Suggett, Sharan Wadhwani, Stephan Ward, Arlo Whithouse, Deborah Wright, Hasan Al Chalabi, Ashish Bhalla, Jo Chmiel, Julie Edmonds, Jodie Fitzgerald, Nicole Isitt, Jonathan Lund, Nicole Mckee, Joely Morgan, Elizabeth Nadin, Ellie Piggott, Rajeev Singh, Katherine Smith, William Speake, Peter Thurley, Samson Tou, Christ Worth, Jill Cooke, Rachel Plummer, Baljit Singh, Ratan Verma, Ndkeita Barnett, Adrian Butler, Susan Gallagher, Amanda Hall, Kar Wai Lau, Mia Marsden, Michael Martin, Katrina Parkinson, Rochelle Rhodes, Alison Tilley, Janine Mallinson, Tania Neale, Ian Renwick, Jacqui Smith, Alison Turnbull

## Abstract

**Background:**

Around 15% of people with colon cancer present with an obstruction. Stenting is appropriate for patients unfit for surgery and/or those with advanced cancer. Patients are living longer with advanced colon cancer; stent design (covered *versus* uncovered) may influence stent re-intervention and quality of life (QoL).

**Methods:**

CReST2 is a phase III multicentre RCT. Patients were randomized 1 : 1 to receive either a covered or uncovered stent. Patients and all medical personnel except the person placing the stent were blinded to allocation. Treatment allocation was via a central randomization service, minimized for: age (≤70 years, >70 years), WHO performance status, tumour site, and indication for palliation. Co-primary endpoints were stent patency up to 6 months after randomization and QoL at 3 months (30 days for patients who died before 3 months) from randomization measured using the European Organisation for Research and Treatment of Cancer (EORTC) QLQ-C30 global health score. Secondary endpoints were stenting success rate, rates of short-term (30 days), intermediate-term (1–3 months), and long-term (3–6 months) stent-related complications, stent-related complication rates of patients undergoing chemotherapy within 6 months after randomization, cumulative frequency of stoma formation, survival at 6 months, and overall survival.

**Results:**

A total of 377 patients were randomised across 28 sites, in whom stenting was unsuccessful in 47 (12.5%) patients (27 of 188: 14.4% covered and 20 of 186: 10.7% uncovered stents). Stent patency at 6 months in stented patients was 117 of 161 (72.7%, covered) and 136 of 166 (81.9%, uncovered) (adjusted HR 1.48, 97.5% confidence interval (c.i.): 0.86–2.54). In this stented population, 216 patients (66.1%) contributed to QoL assessment at 3 months with mean(s.d.) QLQ-C30 global health scores of 54.1(23.9) and 51.6(25.4) in the covered and uncovered groups respectively (adjusted mean difference 1.63, 97.5% c.i. –5.85–9.11). The total numbers of patients experiencing at least one complication in the first 6 months after randomization were 42 of 161 (26.1%) for covered stents and 29 of 166 (17.5%) for uncovered stents. Stent migration was the most common complication and was higher in the covered group. In the covered group and the uncovered group, 44 of 161 (27.3%) and 40 of 166 (24.1%) patients respectively received chemotherapy up to 6 months after randomization. There was a low risk of late perforation associated with both types of stent.

**Conclusion:**

There appears to be greater prolonged stent patency and less stent failure with uncovered stents. QoL is unaffected by stent design.

**Registration number:**

ISRCTN54834267.

## Teaser

For patients stented for treatment of advanced colon cancer, stent design (covered *versus* uncovered) may influence stent re-intervention and quality of life. CReST2 is a phase III multicentre and blinded RCT. Patients were randomized 1 : 1 (minimized for: age (≤70 years, >70 years), WHO performance status, tumour site, and indication for palliation) to a covered or uncovered stent. Quality of life was not affected by stent design, but there was a trend to less stent failure with uncovered stents.

## Introduction

Despite efforts to increase the rates of early diagnosis, each year around 15% of people with colorectal cancer present with obstruction^1^. In many patients with colorectal cancer, their age, their general health, and the advanced state of their cancer means that they may not withstand emergency surgery. Stenting provides an immediate relief of symptoms, whilst avoiding the need for a stoma.

In the palliative situation, several studies have demonstrated the short-term benefits of stenting compared with emergency surgery^2–5^, although the number of patients in randomized studies is small. These benefits include a reduced 30-day mortality, shorter hospitalization, reduced ICU admission rates, and a shorter time to initiation of chemotherapy. Improvements in chemotherapy and biological therapies have resulted in colorectal cancer patients living longer with a stent *in situ*. Stent-related complications have been reported in over a third of patients and include bowel perforation, stent occlusion, and migration^6–9^, raising concerns that severe complications limit the long-term success of stents^10^.

Two designs of stent are in common use. In the UK, the majority of stents placed are made of bare metal (uncovered stents), the remainder have a plastic covering designed to reduce the risk of the tumour growing into the lumen and causing re-obstruction (covered stents). Non-randomized studies and two small randomized trials^8,11^ have reported that stent design may affect complication rates and re-intervention rates. A systematic review^12^ reported that uncovered stents were associated with a lower late migration rate than covered stents, a higher tumour ingrowth rate, and prolonged stent patency. Studies have included patients treated as a bridge to surgery and patients with metastatic disease from other cancer primaries. No studies have been blinded. As such, the quality of evidence is limited.

CReST2 was designed to determine which stent design, covered or uncovered, is the more efficacious (in terms of stent patency and complications) and better in improving the quality of life (QoL) in patients with bowel obstruction arising from colorectal cancer in the palliative setting.

## Methods

### Study design and participants

This RCT (ISRCTN54834267) took place in 28 acute National Health Service (NHS) hospitals in the UK. Eligible participants were aged >16 years, presenting with left-sided colonic obstruction considered to be due to incurable colonic malignancy. Patients with signs of peritonitis and/or perforation, incipient caecal perforation, or obstruction in the mid or lower rectum, patients being treated with or considered for treatment with antiangiogenic drugs (for example bevacizumab), and pregnant patients were excluded. The patient pathway is shown in *supplementary material S1*.

Participants were assigned in a 1 : 1 ratio to insertion of either a covered stent or an uncovered stent. Randomization was performed by telephone or by a secure online randomization system at the Birmingham Clinical Trials Unit (BCTU) (available at https://www.trials.bham.ac.uk/CReST2). After consent and before stent insertion, all participants completed the baseline QoL questionnaire. A minimization algorithm was used to ensure balance in the treatment allocation over the following variables: age (≤70 years, >70 years), WHO performance status (0, 1, 2, 3, 4), tumour site (ascending colon, hepatic flexure, transverse colon, splenic flexure, descending colon, sigmoid, rectosigmoid, proximal rectum), and indication for palliation (unresectable local disease, unresectable metastatic disease, unfit for surgery).

The trial was double-blinded. Only the trials unit and the clinical specialist responsible for stent insertion were aware of the randomized allocation. The patient, the clinician responsible for follow-up, and all other site personnel remained blinded to the type of stent placed. Unblinding was permissible if required due to an urgent clinical need or patient safety issue. All participants provided written informed consent to participate. For the duration of the study, interim analyses of data on both efficacy and safety were reviewed, in strict confidence, by an independent Data Monitoring and Ethics Committee.

### Procedures

Participating centres must have placed ≥30 stents for the treatment of obstructing colorectal cancer, with participating individual surgeons, radiologists, or endoscopists having placed ≥10. Centres that had placed <30 stents were eligible to participate in CReST2 after review of their stenting data by the CReST2 clinical leads. A standard stent insertion technique was agreed, which included a pre-procedure enema, a combined endoscopic/fluoroscopic technique, involving the endoscopic insertion of a self-expanding metal stent (SEMS) and no pre- or post-dilatation, and a post-procedure plain X-ray^13^.

A team comprising designated lead surgeons, radiologists, and gastroenterologists was formed in each centre to establish a clear management pathway. Patients were classified before randomization as having disease suitable for palliative treatment. After resuscitation, patients allocated to stenting had a SEMS deployed across the tumour. Stenting was considered palliative and no further surgery was mandated.

All patients in CReST2 were allocated to receive one of two different types of stent—uncovered or covered. Stents with any covering were classed as covered stents. The stents used in CReST2 were all existing, commercially available, marketed products, which were licensed and CE marked. Participating trusts could select the stent of their choice from a range of suitable stents approved for use within the trial by the CReST2 Trial Management Group. This allowed practitioners inserting stents to use the stent type with which they were most familiar.

### Outcomes

The first 12 months of recruitment formed an internal feasibility study. The aim of the internal feasibility study was to assess the rate of recruitment at 12 months after the start of recruitment and to assess clinician equipoise, thus enabling the feasibility of the study to be determined. The full phase III part of CReST2 had two co-primary outcomes: QoL at 3 months (30 days for patients who died before 3 months) from randomization measured using the European Organisation for Research and Treatment of Cancer (EORTC) QLQ-C30 global health score and stent patency up to 6 months after randomization. Secondary outcomes were stenting success rate in each group (defined as clinical relief of bowel obstruction), rates of short-, intermediate-, and long-term stent-related complications in each group (30 days, 1–3 months, and 3–6 months after stent insertion respectively), stent-related complication rates of patients on chemotherapy in each group up to 6 months after randomization, cumulative frequency of stoma formation in each group, survival at 6 months in each group, and overall survival in each group; QoL at 3 months was also measured using the EORTC QLQ-CR29 disease-specific module for colorectal cancer. Stent-related complications included, but were not limited to, bowel perforation, stent migration, and re-obstruction. Stent follow-up forms collected data on stent-related complications, stoma presence, and re-interventions. Any serious adverse events (SAEs) that occurred were recorded over the course of the entire follow-up interval.

To investigate the representativeness of the randomized population, centres were asked to record a limited amount of anonymized data on patients with left-sided bowel obstruction presumed secondary to carcinoma who were potentially eligible for the trial, but who were not part of the trial.

### Statistical analysis

The sample size for CReST2 was based on two co-primary outcomes: QLQ-C30 global health score^14^ at 3 months and stent patency at 6 months. For QoL, a 0.5 s.d. difference between groups would be clinically meaningful^15^. For stent patency, the expected patency rate in the control arm was 30% and an improvement to 50% in the patients receiving a covered stent would be clinically meaningful. To detect a difference of 0.5 s.d. in QoL and an improvement in stent patency from 30% to 50% between groups using the standard methods (comparing means and comparing proportions with continuity correction respectively) with 90% power and a type I error rate of 2.5% to account for the multiple comparisons, a total of 157 participants per group would need to be randomized, 314 in total. Assuming and adjusting for a 10% loss to follow-up/dropout rate, target recruitment was 350 participants. The co-primary analyses were undertaken on a modified intention-to-treat (ITT) basis, meaning that the co-primary analyses were restricted to those participants who had a successful first stent insertion procedure after randomization, irrespective of treatment compliance. The covariates used for adjustment for this outcome were the minimization variables—age, WHO performance status, tumour site, and indication for palliation. QoL at 3 months was measured using the QLQ-C30 global health score using the official scoring manual^16^. Using data obtained at the 3-month time point (or 30-day time point if 3-month data were not available), the adjusted mean difference and 97.5% confidence interval was estimated using a linear regression model and a *P* value was calculated. The covariates used for adjustment for this outcome were the baseline global health score, plus the minimization variables.

Stent failure occurred when the stent was no longer providing luminal patency. The time to stent failure up to 6 months after randomization was compared between groups using an adjusted Cox proportional hazards model and is displayed in a Kaplan–Meier plot. The adjusted HR and 97.5% confidence interval was estimated and a *P* value was calculated. Statistical significance of the treatment group parameter was determined from the *P* value generated by the model.

Two sensitivity analyses were performed to test the robustness of the co-primary QoL analysis: including all randomized participants (excluding the 3 complete withdrawals) regardless of the success of their initial stenting procedure; and restricting the analysis to those participants with successful stent insertions who were adherent to their randomized allocation. Similarly, one sensitivity analysis was performed to test the robustness of the co-primary stent patency analysis, restricting the analysis to those participants with successful initial stent insertions who were adherent to their randomized allocation. The adjusted mean differences/HRs, 97.5% confidence intervals, and *P* values were calculated. The covariates used for adjustment for these outcomes were the minimization variables.

Subgroup analyses were limited to the two co-primary outcomes only and the four variables for which the randomization was minimized: age (≤70 years, >70 years), WHO performance status (0, 1, 2, 3, 4), tumour site (right colon, left colon/rectum), and indication for palliation (unresectable local disease, unresectable metastatic disease, unfit for surgery). The effects of these subgroups were examined by including a treatment group by subgroup interaction parameter in the original regression models for the co-primary analyses. The *P* values for the interaction and the adjusted mean differences/HRs and 97.5% confidence intervals were calculated. A forest plot was constructed for visual representation of the data for each co-primary outcome. The results of subgroup analyses were interpreted with caution and considered as hypothesis-generating only.

Secondary time-to-event outcomes were analysed using Cox proportional hazard models, as per the co-primary outcome. Secondary binary outcomes were analysed using log-binomial models or, in cases of non-convergence, using Poisson models with robust standard errors. As per the co-primary analyses, 97.5% confidence intervals were calculated. For binary outcomes, both absolute and relative effect sizes are presented.

For all analyses, *P* values are two-sided and *P* = 0.025 was considered significant. Analyses were performed using SAS^®^ version 9.4 (SAS Institute, Cary, NC, USA) and Stata version 18.0.

## Results

**Fig. 1:**
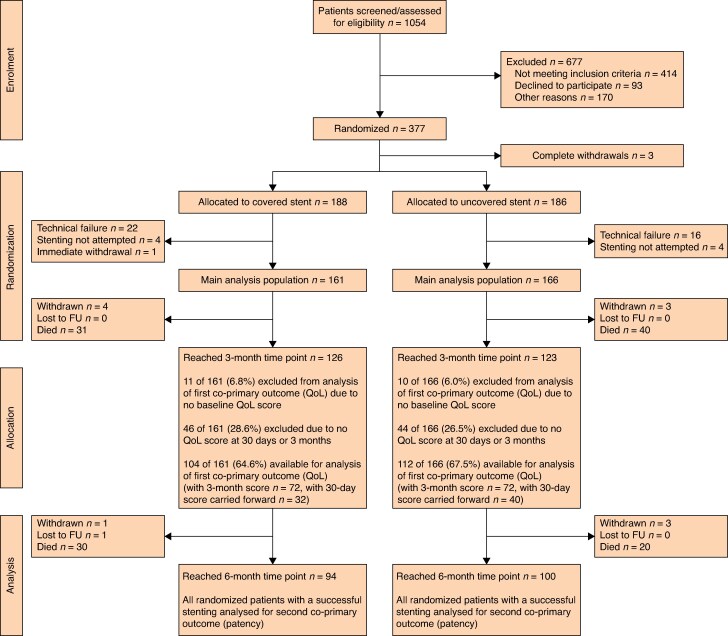
CONSORT diagram FU, follow-up; QoL, quality of life.

Between 12 June 2017 and 27 April 2022, 1054 patients were assessed for eligibility; 640 (60.7%) were considered eligible. In total, 377 patients with obstructing colorectal cancer treated with palliative intent were randomized between covered and uncovered stents; 3 patients completely withdrew from the study, leaving 374 patients for analysis (*Fig. 1*).^17^ The database was locked for analysis on 27 June 2023, when all participants had reached the 6-month assessment and the corresponding outcome data had been entered onto the study database and validated as being ready for analysis.

Baseline characteristics were balanced across treatment arms (*Table 1*). The mean age at randomization was 73.7 (range 27–98) years, 63.6% were men, 69.8% had WHO performance status 0 or 1 (fully active or mobile all day), and almost half (48.1%) had a sigmoid tumour. The main indication for palliation was unresectable metastatic disease (49.5%), with the remainder being evenly split between unresectable local disease (25.4%) and considered unfit for surgery (25.1%). Of the patients, 93.3% were diagnosed using CT and 7.5% were diagnosed using MRI. Metastases were present in 71.9% of participants. Baseline characteristics for the modified ITT analysis data set were also balanced (*supplementary material S2*).

**Table 1:** Baseline characteristics

	Covered stent *n* = 188	Uncovered stent *n* = 186	Overall *n* = 374
**Minimization variables**			
Age (years)			
≤70	67 (35.6%)	62 (33.3%)	129 (34.5%)
>70	121 (64.4%)	124 (66.7%)	245 (65.5%)
WHO performance status			
0: fully active	57 (30.3%)	55 (29.6%)	112 (30.0%)
1: mobile all day	73 (38.8%)	76 (40.9%)	149 (39.8%)
2: in bed <50%	40 (21.3%)	39 (21.0%)	79 (21.1%)
3: in bed ≥50%	16 (8.5%)	14 (7.5%)	30 (8.0%)
4: bedridden	2 (1.1%)	2 (1.1%)	4 (1.1%)
Primary tumour site			
Ascending colon	4 (2.1%)	3 (1.6%)	7 (1.8%)
Hepatic flexure	5 (2.7%)	6 (3.2%)	11 (2.9%)
Transverse colon	16 (8.5%)	15 (8.1%)	31 (8.3%)
Splenic flexure	14 (7.5%)	15 (8.1%)	29 (7.8%)
Descending colon	23 (12.2%)	24 (12.9%)	47 (12.6%)
Sigmoid	89 (47.3%)	91 (48.9%)	180 (48.1%)
Rectosigmoid	28 (14.9%)	25 (13.4%)	53 (14.2%)
Rectum (proximal)	9 (4.8%)	7 (3.8%)	16 (4.3%)
Indication for palliation			
Unresectable local disease	47 (25.0%)	48 (25.8%)	95 (25.4%)
Unresectable metastatic disease	93 (49.5%)	92 (49.5%)	185 (49.5%)
Considered unfit for surgery	48 (25.5%)	46 (24.7%)	94 (25.1%)
**Demographic and other baseline variables**			
Age (years)			
Mean (SD)	73.0 (15.0)	74.5 (13.2)	73.7 (14.1)
Range	27.0–98.0	32.0–98.0	27.0–98.0
Missing	0	0	0
Sex ratio (M : F)	126 : 62	112 : 74	238 : 136
Method of diagnosis*			
Colonoscopy	66 (35.1%)	77 (41.4%)	143 (38.2%)
CT scan	177 (94.2%)	172 (92.5%)	349 (93.3%)
Flexible sigmoidoscopy	56 (29.8%)	53 (28.5%)	109 (29.1%)
MRI	14 (7.5%)	14 (7.5%)	28 (7.5%)
Metastases diagnosed			
No	55 (29.3%)	49 (26.3%)	104 (27.8%)
Yes	132 (70.2%)	137 (73.7%)	269 (71.9%)
Missing	1 (0.5%)	0 (0.0%)	1 (0.3%)

^*^Note that more than one method of diagnosis can be specified; therefore, the sum of the numbers presented may exceed the total number of randomized patients on the trial.

The stenting success rate (defined as clinical relief of bowel obstruction) was 161 of 188 (85.6%) for covered stents and 166 of 186 (89.2%) for uncovered stents (adjusted incidence rate ratio 0.96 (97.5% c.i. 0.88 to 1.05); adjusted risk difference −0.03 (97.5% c.i. −0.10 to 0.05)) (*Table 2*). Technical failure of initial stent insertion occurred in 22 of 188 (11.7%) of the covered stent group *versus* 16 of 186 (8.6%) of the uncovered stent group. The most common cause of stent technical failure was an inability to pass a guidewire into the colon proximal to the obstruction. Additionally, there were four patients in each group where stent insertion was not attempted and one post-randomization withdrawal in the covered stent group. Further details on the stent insertion procedure can be found in *supplementary material S3*.

**Table 2:** Technical success and primary outcome results

	Covered stent *n* = 188	Uncovered stent *n* = 186	Estimate (97.5% CI)	*P*-value
**Was stent insertion technically successful?**				
** Yes**	**161 (85.6%)**	**166 (89.2%)**		
** No/not stented/form missing**	**27 (14.4%)**	**20 (10.8%)**		
Unadjusted Poisson model (IRR)*			0.96 (0.88, 1.05)	*P* = 0.293
Unadjusted log-binomial model with identity link (risk difference)			−0.04 (−0.11, 0.04)	*P* = 0.291
Adjusted Poisson model (IRR)†			0.96 (0.88, 1.05)	*P* = 0.260
Adjusted log-binomial model with identity link (risk difference)			−0.03 (−0.10, 0.05)	*P* = 0.482
**Reasons for lack of technical success** ‡				
Technical failure	22 (11.7%)	16 (8.6%)		
Stent not attempted	4 (2.1%)	4 (2.2%)		
Post-randomization withdrawal	1 (0.5%)	0 (–)		
**QLQ-C30 global health score**				
Technically successful, with a baseline global health score, AND at either 30 days or at 3 months post randomization	104	112		
3 month score available	*72*	*72*		
30 day score carried forward	*32*	*40*		
Global health score at baseline	48.7 (23.7)	47.0 (26.5)		
Global health score at month 3, mean(s.d.)§	54.1 (23.9)	51.6 (25.4)	2.13 (−5.27, 9.54)	0.516¶
			1.60 (−6.05, 9.24)	0.637#
			1.63 (−5.85, 9.11)	0.624**
**Stent failure by 6 months**				
No (patent)	117 (72.7%)	136 (81.9%)		
Yes (failure)	44 (27.3%)	30 (18.1%)		
Unadjusted Cox proportional hazards model (HR)			1.56 (0.92, 2.65)	0.060
Adjusted Cox proportional hazards model (HR)††			1.48 (0.86, 2.54)	0.104

Values are *n* (%) unless otherwise indicated.

^*^A log-binomial model failed to converge; a Poisson regression model with robust standard errors was used to estimate the unadjusted parameters. An incidence rate ratio <1 favours the uncovered group.

^†^A log-binomial model failed to converge; a Poisson regression model with robust standard errors was used, adjusting for age, WHO performance status, tumour site, and indication for palliation, where tumour-site variable was collapsed into right colon (ascending colon, hepatic flexure, transverse colon, splenic flexure) and left colon/rectum (descending colon, sigmoid, rectosigmoid, rectum (proximal)). An incidence rate ratio <1 favours the uncovered group.

^‡^More than one reason could be selected; therefore, the sum of the column numbers may exceed the total number.

^§^Higher mean scores indicate higher QoL. A positive difference favours the covered group. Missing values for global health score at month 3 have been imputed using the corresponding score at 30 days where available.

^¶^Mean difference (covered − uncovered) calculated using a linear regression model adjusted for baseline global health score.

^#^Mean difference (covered − uncovered) calculated using a linear regression model, adjusting for baseline global health score, age, WHO performance status, tumour site, and indication for palliation.

^**^As in (#) but tumour site variable collapsed into right colon (ascending colon, hepatic flexure, transverse colon, splenic flexure) and left colon/rectum (descending colon, sigmoid, rectosigmoid, rectum (proximal)).

^††^Cox proportional hazards model, adjusting for age, tumour site, WHO performance status, and indication for palliation. Tumour site was collapsed into right colon (ascending colon, hepatic flexure, transverse colon, splenic flexure) and left colon/rectum (descending colon, sigmoid, rectosigmoid, rectum (proximal)). A hazard ratio <1 favours the covered group. IRR, incidence rate ratio; QLQ-C30, Questionnaire designed to assess the QoL of cancer patients.

All those patients who did not have technically successful stent insertions were deemed non-adherent to their allocation (27 of 188 patients in the covered stent group *versus* 20 of 186 patients in the uncovered stent group). Of those who had technically successful stent insertions, six patients in the covered group and two patients in the uncovered group were known to be non-adherent to their allocation; information on stent design was not available for a further two patients in the covered group and three patients in the uncovered group and these patients were assumed to be non-adherent. This resulted in an overall adherence rate of 153 of 188 (81.4%) in the covered group and 161 of 186 (86.6%) in the uncovered group. Restricting the calculation to those with technically successful stent insertions (the modified ITT analysis population) gave adherence rates of 153 of 161 (95.0%) *versus* 161 of 166 (97.0%) respectively.

The crude health-related QoL measured using EORTC QLQ-C30 at 3 months after stent insertion was higher than that reported at baseline for both groups. This improvement was maintained for 24 months after stent insertion (*supplementary material S5*). However, there was no difference in health-related QoL between the covered and uncovered stent groups for those patients successfully stented (modified ITT), adjusting for baseline global health score, age, WHO performance status, tumour site, and indication for palliation (adjusted mean difference 1.63 (97.5% c.i. −5.85 to 9.11), adjusted *P* = 0.624) (*Table 2*). Subgroup analyses did not identify any characteristics that favoured a covered or uncovered stent (*supplementary material S4*).

**Fig. 2:**
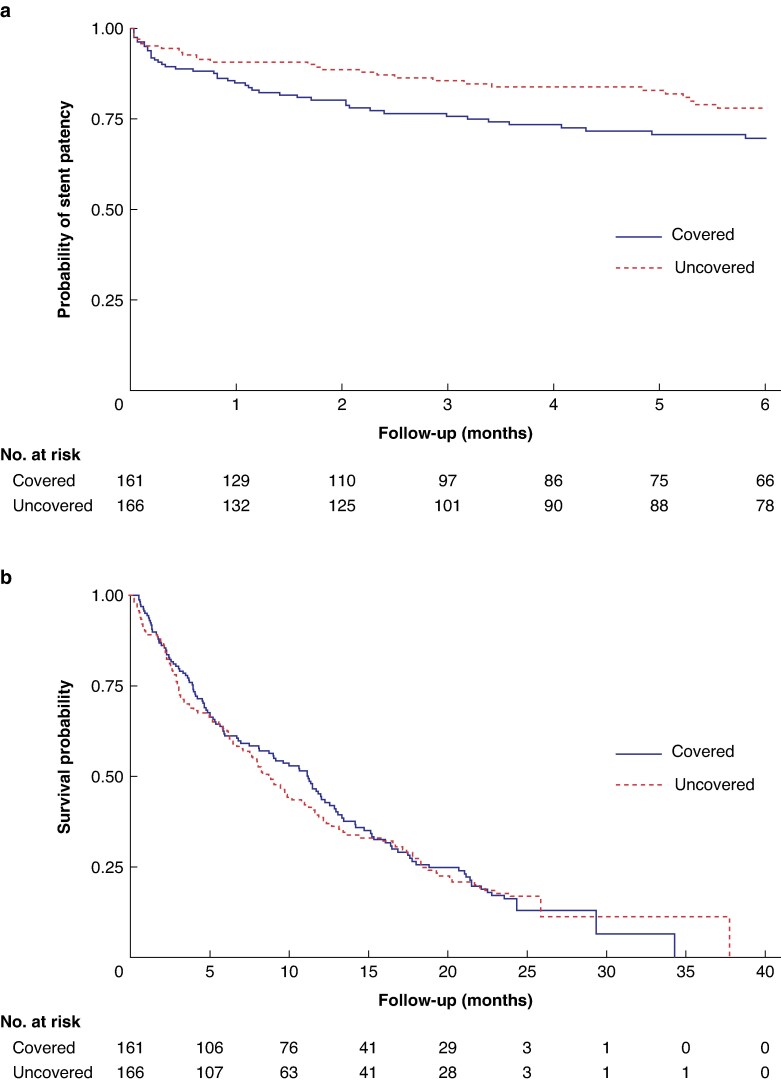
Kaplan–Meier plots **a** Kaplan–Meier plot of time to first stent failure up to 6 months (for patients who had a technically successful first stent insertion). **b** Kaplan–Meier plot of time to death up to the end of follow-up (for patients who had a technically successful first stent insertion).

There was a trend towards fewer stent failures with uncovered stents, adjusting for age, tumour site, WHO performance status, and indication for palliation (HR 1.48 (97.5% c.i. 0.86 to 2.54), adjusted *P* = 0.104) (*Table 2* and *Fig. 2a*). The difference was largely due to less stent migration (*Table 3*). The subgroup analyses of stent patency showed that there may be some difference between the age subgroups (≤70 years *versus >*70 years); in the group of patients aged >70 years stent failure was more likely to occur for covered stents (test for interaction *P* = 0.028). There were no differences seen in the other subgroup analyses for the stent patency outcome (*supplementary material S6*).

**Table 3:** Stent-related complications

	Covered stent (*n* = 161)		Uncovered stent (*n* = 166)	
**All patients with a technically successful stent insertion** *				
Stent migration				
30 days	15 (9.3%)		7 (4.2%)	
1–3 months	9 (5.6%)		4 (2.4%)	
3–6 months	2 (1.2%)		1 (0.6%)	
Number of patients experiencing at least one migration across the three time points	26 (16.2%)		12 (7.2%)	
Re-obstruction				
30 days	5 (3.1%)		4 (2.4%)	
1–3 months	4 (2.5%)		2 (1.2%)	
3–6 months	3 (1.9%)		6 (3.6%)	
Number of patients experiencing at least one re-obstruction across the three time points	12 (7.5%)		11 (6.6%)	
Perforation				
30 days	6 (3.7%)		8 (4.8%)	
1–3 months	2 (1.2%)		1 (0.6%)	
3–6 months	3 (1.9%)		0 (0.0%)	
Number of patients experiencing at least one perforation across the three time points	11 (6.8%)		9 (5.4%)	
Combined	Patients *n* (%)	Events *n*	Patients *n* (%)	Events *n*
30 days	23 (14.3)	26	16 (9.6)	19
1–3 months	13 (8.1)	15	7 (4.2)	7
3–6 months	8 (5.0)	8	7 (4.2)	7
Total		49		33
Number of patients experiencing at least one complication across the three time points	42 (26.1%)		29 (17.5%)	
	**Covered stent (*n* = 44)**		**Uncovered stent (*n* = 40)**	
**All patients with a technically successful stent insertion who received chemotherapy at any point up to 6 months** *				
Stent migration				
30 days	4 (9%)		2 (5%)	
1–3 months	4 (9%)		1 (3%)	
3–6 months	2 (5%)		1 (3%)	
Number of patients experiencing at least one migration across the three time points	10 (23%)		4 (10%)	
Re-obstruction				
30 days	1 (2%)		1 (3%)	
1–3 months	2 (5%)		0 (0%)	
3–6 months	0 (0%)		3 (8%)	
Number of patients experiencing at least one re-obstruction across the three time points	3 (7%)		4 (10%)	
Perforation				
30 days	0 (0%)		3 (8%)	
1–3 months	0 (0%)		1 (3%)	
3–6 months	1 (2%)		0 (0%)	
Number of patients experiencing at least one perforation across the three time points	1 (2%)		4 (10%)	
Combined	Patients *n* (%)	Events *n*	Patients *n* (%)	Events *n*
30 days	4 (9)	5	5 (13)	6
1–3 months	5 (11)	6	2 (5)	2
3–6 months	3 (7)	3	4 (10)	4
Total		14	12	
Number of patients experiencing at least one complication across the three time points	11 (25%)		11 (28%)	
	**Covered stent (*n* = 161)**		**Uncovered stent (*n* = 166)**	
**All patients with a technically successful stent insertion who had a recurrent bowel obstruction at any point up to 6 months†**				
Total	38 (23.6%)		29 (17.5%)	
Reasons for stent failure				
Stent overgrowth	5 (13%)		3 (10%)	
Stent ingrowth	4 (11%)		5 (17%)	
Stent migration	24 (63%)		13 (45%)	
Stent perforation	7 (18%)		6 (21%)	
Other	8 (21%)		7 (24%)	

Values are *n* (%) unless otherwise indicated.

^*^At any time point, a patient can only have up to one event recorded for each type of the three complications.

^†^Multiple reasons may be given for a stent failure, so the total percentage value may be >100.

Two sensitivity analyses were performed for the co-primary QoL outcome. The analysis that included all randomized participants regardless of technical success showed an adjusted mean difference of 1.76 (97.5% c.i. −5.46 to 8.98) and an adjusted *P* value of 0.583. The analysis that was restricted to those participants adherent to their randomized allocation showed a mean difference of 1.91 (97.5% c.i. −5.70 to 9.53) and an adjusted *P* value of 0.571. The sensitivity analysis for the co-primary stent patency outcome, restricting the analysis population to the adherent patients, showed an HR of 1.52 (97.5% c.i. 0.87 to 2.66) and an adjusted *P* value of 0.093. These results are in agreement with those reported for the co-primary analyses (*supplementary material S7*).

The time to onset and nature of stent-related complications are shown in *Table 3*. The majority of SAEs occurred during the first 30 days after insertion. The most important SAE, perforation, occurred in 11 of 161 (6.8%) patients in the covered group and 9 of 166 (5.4%) patients in the uncovered group. The total number of patients experiencing at least one SAE at any point between randomization and 6 months was 42 of 161 (26.1%) for covered stents and 29 of 166 (17.5%) for uncovered stents (chi-squared test *P* = 0.059). Stent migration was the most common SAE and was higher in the covered stent group, 26 of 161 (16.2%) *versus* 12 of 166 (7.2%) (chi-squared test *P* = 0.012). The rates and nature of stent-related complications for patients receiving chemotherapy are also shown in *Table 3*. Only a quarter of patients received chemotherapy during the 6 months after randomization. Similar complication rates were noted in both groups. The feared complication of large bowel perforation during chemotherapy was rare; only one patient in each group developed perforation >30 days after stent insertion.

Endoscopic re-intervention up to 6 months after randomization was attempted in 13 of 161 (8.1%) covered stent patients and 6 of 166 (3.6%) uncovered stent patients (adjusted risk ratio 2.08 (97.5% c.i. 0.70 to 6.19), adjusted *P* = 0.132; adjusted risk difference 0.04 (97.5% c.i. −0.01 to 0.09), adjusted *P* = 0.118). Stoma rates were low, with rates of 11 of 161 (6.8%) for covered stents and 11 of 166 (6.6%) for uncovered stents (adjusted risk ratio 0.91 (97.5% c.i. 0.36 to 2.29), adjusted *P* = 0.813; adjusted risk difference −0.01 (97.5% c.i. −0.06 to 0.05), adjusted *P* = 0.814).

Survival until withdrawal from the trial or at 6 months was 100 of 161 (62.1%) for covered stent patients and) 106 of 166 (63.9%) for uncovered stent patients (HR 0.98 (97.5% c.i. 0.65 to 1.47), adjusted *P* = 0.894). Overall survival was 40 of 161 (24.8%) for covered stent patients *versus* 42 of 166 (25.3%) for uncovered stent patients. A Kaplan–Meier plot for survival is shown in *Fig. 2b*.

QoL at 3 months measured using the EORTC QLQ-CR29 disease-specific module for colorectal cancer did not show any differences between the two groups (*supplementary material S8*).

The number and type of reported SAEs that were related to treatment were broadly balanced between those receiving uncovered and covered stents. An apparent increase in colonic ulcers was seen (19 covered *versus* 10 uncovered). Without differences in pain or perforation, the presence of colonic mucosal ulceration in likely of no significance.

## Discussion

Stenting in the palliative setting can achieve high procedural and clinical success rates. Clinical success was not statistically significantly different between groups—85.6% in the covered stent group and 89.2% in uncovered stent group—giving an overall rate of 87.4%, which is comparable to the 86.1% clinical success rate reported in systematic reviews^18^.

After stent insertion, QoL improved and was maintained for 24 months in those surviving for that length of time. No evidence that QoL was influenced by stent design was found, although the possibility of small differences cannot be ruled out. When considered with the high clinical success rate, this finding lends strong support for the use of stenting in the palliative setting.

Mol *et al*.^19^ considered an EORTC QLQ-C30 score of <66.7% to indicate poor performance status. The median overall survival for those with a poor performance status was 10.4 months^19^. Patients in CReST2 consistently reported EORTC QLQ-C30 scores below this level, consistent with advanced disease at presentation.

Comparison of data from the present trial with data from previous trials is impaired by the small number of patients in randomized trials, different indications for stent treatment (bridge to surgery *versus* palliation), different patient populations (colorectal cancer *versus* extrinsic metastatic disease from other primary sites), and the potential bias inherent in non-randomized studies without blinding. A total of 144 patients treated with palliative intent have been reported in previous RCTs and 41 of these did not have colorectal cancer (28.5%)^8,11^. Therefore, CReST2 contributes to the understanding of this treatment pathway.

Of those with an uncovered stent, 81.9% of stents remained patent until withdrawal/death or at 6 months and only 6.6% required a stoma. On a modified ITT basis, there was a trend to improved stent patency at 6 months in the uncovered stent group. Subgroup analysis additionally favoured uncovered stents for patients aged >70 years. Maintenance of stent patency has been previously reported to vary considerably from 53% to 90% until death or end of follow-up^20–22^. This wide variation may reflect patient heterogeneity, but more likely it relates to technical failures. CReST2 is associated with extensive training and cross-site learning, which is reflected in the positive outcomes reported.

The total number of patients who experienced at least one stent-related complication in the present trial was lower than the 34.0% reported in a meta-analysis of ten non-randomized and three randomized studies^23^. The principal stent-related complications are perforation, migration, and re-obstruction. In CReST2, the rate of perforation was not different between the groups (perforation occurred in 6.8% of patients with covered stents and 5.4% of patients with uncovered stents). An overall perforation rate of 7.4% was reported in a meta-analysis of 4086 patients^24^.

Stent migration was the most common complication. In the covered stent group, migration occurred more than twice as frequently as in the uncovered stent group. This is similar to previous reports, in the randomized study by Park *et al*; 21.1% for covered stents and 1.8% for uncovered stents^8^ and higher in three previous systematic reviews^7,12,25^.

Covered stents were developed to reduce the risk of stent occlusion caused by tumour ingrowth. Increased tumour ingrowth has been reported with uncovered stents^7,8,12^, but this does not appear to worsen long-term patency. Stent occlusion, due to overgrowth of malignant tissue at the top and bottom of the stent, tumour ingrowth, and faecal impaction, occurred at a rate of 18% for all stent types^23^. In CReST2, stent blockage occurred at the lower end of the reported range and, importantly, no difference in rates of stent blockage was observed.

Endoscopic re-intervention was attempted in 8.1% of covered stent patients and 3.6% of the uncovered stent group. Endoscopic re-intervention by stent-in-stent placement was clinically successful in 75% of cases with a median duration of patency of 170 days^26^ and is recommended in European guidelines^27^. Concerns remain that the risk of colonic perforation is increased in ‘stented patients’ undergoing chemotherapy. In CReST2, a quarter of patients received chemotherapy during the 6 months after randomization. The feared complication of large bowel perforation during chemotherapy was rare; only one patient in each group developed perforation >30 days after stent insertion. A review of the literature in 2022 reported on 13 retrospective studies and one randomized trial that contributed only nine patients to the study. In the 682 patients included, chemotherapy was not associated with a higher risk of stent-related complications^28^.

## Collaborators

James Hill (Manchester University NHS Foundation Trust, Manchester, UK), Nicola Fearnhead (Cambridge University Hospitals NHS Foundation Trust, Cambridge, UK), Richard Gray (University of Oxford, Oxford, UK), Kelly Handley (University of Birmingham, Birmingham, UK), Manjinder Kaur (University of Birmingham, Birmingham, UK), Clive Kay (Kings College Hospital NHS Foundation Trust, London, UK), Hans-Ulrich Laasch (The Christie NHS Foundation Trust, Manchester, UK), Andrew Lowe (Taunton and Somerset NHS Foundation Trust, Somerset, UK), Laura Magill (University of Birmingham, Birmingham, UK), Dion Morton (University Hospitals Birmingham NHS Foundation Trust, Birmingham, UK), Ruben Mujica-Mota (University of Leeds, Leeds, UK), Andy Palmer (University of Birmingham, Birmingham, UK), Anne Pullyblank (North Bristol NHS Trust, Bristol, UK), Yongzhong Sun (University of Birmingham, Birmingham, UK), Suresh Vasan Venkatachalapathy (Nottingham University Hospitals NHS Trust), Pete Wheatstone (Patient Represenative), Yasmin Ali (University of Birmingham, Birmingham, UK), Altus Chan (University of Birmingham, Birmingham, UK), Suzanne Locker (University of Birmingham, Birmingham, UK), Paul Riley (University of Birmingham, Birmingham, UK), Gordon Carlson (Retired), Louise Hiller (University ofWarwick, Warwick, UK), Stuart Taylor (University College London Hospitals NHS Foundation Trust, London, UK), Sarah Barry (University of Strathclyde, Scotland, UK), Philip Bell (Patient Representative), Steve Halligan (University College London Hospitals NHS Foundation Trust, London, UK), Nigel Scott (Retired), Suhail Ahmed (Aintree University Hospitals NHS Foundation Trust, Liverpool, UK), James Arthur (Aintree University Hospitals NHS Foundation Trust, Liverpool, UK), Carol Brooks (Aintree University Hospitals NHS Foundation Trust, Liverpool, UK), Jane L Hughes (Aintree University Hospitals NHS Foundation Trust, Liverpool, UK), Tanya Ingram (Aintree University Hospitals NHS Foundation Trust, Liverpool, UK), Michelle Linforth (Aintree University Hospitals NHS Foundation Trust, Liverpool, UK), Sophie Marsh (Aintree University Hospitals NHS Foundation Trust, Liverpool, UK), Rizwan Saleem (Aintree University Hospitals NHS Foundation Trust, Liverpool, UK), Simone Slawik (Aintree University Hospitals NHS Foundation Trust, Liverpool, UK), Sarah Stevenson (Aintree University Hospitals NHS Foundation Trust, Liverpool, UK), Lilian Wajero(Aintree University Hospitals NHS Foundation Trust, Liverpool, UK), Nicholas Cross (Aneurin Bevan University Health Board, Wales, UK), Amanda Dell (Aneurin Bevan University Health Board, Wales, UK), Mandy Edwards (Aneurin Bevan University Health Board, Wales, UK), Angela Hall (Aneurin Bevan University Health Board, Wales, UK), Helen Hamilton (Aneurin Bevan University Health Board, Wales, UK), Nancy Hawkins (Aneurin Bevan University Health Board, Wales, UK), Heidi Lawson (Aneurin Bevan University Health Board, Wales, UK), Mark Robinson (Aneurin Bevan University Health Board, Wales, UK), Michelle Tayler (Aneurin Bevan University Health Board, Wales, UK), Rebecca Wallace (Aneurin Bevan University Health Board, Wales, UK), Sarah Wheatman (Aneurin Bevan University Health Board, Wales, UK), Joanna Wilson (Aneurin Bevan University Health Board, Wales, UK), Lindianne Aitken (Aneurin Bevan University Health Board, Wales, UK), Rhodri Codd (Aneurin Bevan University Health Board, Wales, UK), Joseph Hamill (Aneurin Bevan University Health Board, Wales, UK), Nancy Hawkins (Aneurin Bevan University Health Board, Wales, UK), Georgia Mallison (Aneurin Bevan University Health Board, Wales, UK), Steve McKain (Aneurin Bevan University Health Board, Wales, UK), Heeam Nassa (Aneurin Bevan University Health Board, Wales, UK), Claire Louise Price (Aneurin Bevan University Health Board, Wales, UK), Mark Robinson (Aneurin Bevan University Health Board, Wales, UK), Brian Stephenson (Aneurin Bevan University Health Board, Wales, UK), Keshav Swarnkar (Aneurin Bevan University Health Board, Wales, UK), Claire Triscott (Aneurin Bevan University Health Board, Wales, UK), Elaine Wall (Aneurin Bevan University Health Board, Wales, UK), Rebecca Wallace (Aneurin Bevan University Health Board, Wales, UK), Gethin Williams (Aneurin Bevan University Health Board, Wales, UK), Cerian Williams (Aneurin Bevan University Health Board, Wales, UK), Rommel Butawan (Barking, Havering and Redbridge University Hospitals NHS Trust, Essex, UK), Joseph Huang (Barking, Havering and Redbridge University Hospitals NHS Trust, Essex, UK), Sam King (Barking, Havering and Redbridge University Hospitals NHS Trust, Essex, UK), Tina Mills-Baldock (Barking, Havering and Redbridge University Hospitals NHS Trust, Essex, UK), Purushothaman Premchand (Barking, Havering and Redbridge University Hospitals NHS Trust, Essex, UK), Alison Ray (Barking, Havering and Redbridge University Hospitals NHS Trust, Essex, UK), Amy Barnett (Blackpool Teaching Hospitals NHS Foundation Trust, Blackpool, UK), Alexander Blackmore (Blackpool Teaching Hospitals NHS Foundation Trust, Blackpool, UK), Oliver Brennan (Blackpool Teaching Hospitals NHS Foundation Trust, Blackpool, UK), Melaine Caswell (Blackpool Teaching Hospitals NHS Foundation Trust, Blackpool, UK), Greta Van Doyvenvoorde (Blackpool Teaching Hospitals NHS Foundation Trust, Blackpool, UK), James Glen (Blackpool Teaching Hospitals NHS Foundation Trust, Blackpool, UK), Shamina Hankinson (Blackpool Teaching Hospitals NHS Foundation Trust, Blackpool, UK), Mark Hendrickse (Blackpool Teaching Hospitals NHS Foundation Trust, Blackpool, UK), Peter Isaacs (Blackpool Teaching Hospitals NHS Foundation Trust, Blackpool, UK), Ilianna Mamali (Blackpool Teaching Hospitals NHS Foundation Trust, Blackpool, UK), Senthil Murugesan (Blackpool Teaching Hospitals NHS Foundation Trust, Blackpool, UK), Marina Oprea (Blackpool Teaching Hospitals NHS Foundation Trust, Blackpool, UK), Chris Pemberton (Blackpool Teaching Hospitals NHS Foundation Trust, Blackpool, UK), Ella Riedel (Blackpool Teaching Hospitals NHS Foundation Trust, Blackpool, UK), Arunan Sivapataham (Blackpool Teaching Hospitals NHS Foundation Trust, Blackpool, UK), Wei Fen Tay (Blackpool Teaching Hospitals NHS Foundation Trust, Blackpool, UK), Lauren Thornborough (Blackpool Teaching Hospitals NHS Foundation Trust, Blackpool, UK), Rachel Wheeldon (Blackpool Teaching Hospitals NHS Foundation Trust, Blackpool, UK), Conor Wilkinson (Blackpool Teaching Hospitals NHS Foundation Trust, Blackpool, UK), Julie Chadwick (Bolton NHS Foundation Trust, Bolton, UK), Shirley Cocks (Bolton NHS Foundation Trust, Bolton, UK), Gemma Faulkner (Bolton NHS Foundation Trust, Bolton, UK), Robert Hull (Bolton NHS Foundation Trust, Bolton, UK), Marta Martinez Iglesias (Bolton NHS Foundation Trust, Bolton, UK), James Lay (Bolton NHS Foundation Trust, Bolton, UK), Ha Phuong Do Le (Bolton NHS Foundation Trust, Bolton, UK), James Pollard (Bolton NHS Foundation Trust, Bolton, UK), Shenraga Kumar Rajamanickam (Bolton NHS Foundation Trust, Bolton, UK), Rubeena Razzaq (Bolton NHS Foundation Trust, Bolton, UK), Michaela Sutherland (Bolton NHS Foundation Trust, Bolton, UK), Aphan Abdulholim (Bradford Teaching Hospitals NHS Foundation Trust Bradford, UK), Conrad Beckett (Bradford Teaching Hospitals NHS Foundation Trust Bradford, UK), Wendy Cardozo (Bradford Teaching Hospitals NHS Foundation Trust Bradford, UK), Carol Firth (Bradford Teaching Hospitals NHS Foundation Trust Bradford, UK), Naeem Jagirdar (Bradford Teaching Hospitals NHS Foundation Trust Bradford, UK), Wendy Jepson (Bradford Teaching Hospitals NHS Foundation Trust Bradford, UK), Sarah Jowett (Bradford Teaching Hospitals NHS Foundation Trust Bradford, UK), Amjad Mohammed (Bradford Teaching Hospitals NHS Foundation Trust Bradford, UK), Sulleman Moreea (Bradford Teaching Hospitals NHS Foundation Trust Bradford, UK), Nicolas Rabb (Bradford Teaching Hospitals NHS Foundation Trust Bradford, UK), Jonathan Robinson (Bradford Teaching Hospitals NHS Foundation Trust Bradford, UK), Sophie Stephenson (Bradford Teaching Hospitals NHS Foundation Trust Bradford, UK), Sarah Tinker (Bradford Teaching Hospitals NHS Foundation Trust Bradford, UK), Ashlea Bucke (Cambridge University Hospitals NHS Foundation Trust, Cambridge, UK), Ewen Cameron (Cambridge University Hospitals NHS Foundation Trust, Cambridge, UK), Nicholas Carroll (Cambridge University Hospitals NHS Foundation Trust, Cambridge, UK), Gareth Corbett (Cambridge University Hospitals NHS Foundation Trust, Cambridge, UK), Nicola Fearnhead (Cambridge University Hospitals NHS Foundation Trust, Cambridge, UK), Nigel Hall (Cambridge University Hospitals NHS Foundation Trust, Cambridge, UK), Alisa Liddle (Cambridge University Hospitals NHS Foundation Trust, Cambridge, UK), Ines Modolell (Cambridge University Hospitals NHS Foundation Trust, Cambridge, UK), Jonathan Morton (Cambridge University Hospitals NHS Foundation Trust, Cambridge, UK), Aileen Nacorda (Cambridge University Hospitals NHS Foundation Trust, Cambridge, UK), Sophie Newton (Cambridge University Hospitals NHS Foundation Trust, Cambridge, UK), Beverley Nobbs (Cambridge University Hospitals NHS Foundation Trust, Cambridge, UK), Debbie Read (Cambridge University Hospitals NHS Foundation Trust, Cambridge, UK), Rebekka Troller (Cambridge University Hospitals NHS Foundation Trust, Cambridge, UK), James Wheeler (Cambridge University Hospitals NHS Foundation Trust, Cambridge, UK), Lucy Worboys (Cambridge University Hospitals NHS Foundation Trust, Cambridge, UK), Megan Brickhill (Manchester University NHS Foundation Trust, Manchester, UK), Chris Craig (Manchester University NHS Foundation Trust, Manchester, UK), Finlay Curran (Manchester University NHS Foundation Trust, Manchester, UK), David Donnelly (Manchester University NHS Foundation Trust, Manchester, UK), Mona Fareh (Manchester University NHS Foundation Trust, Manchester, UK), Bethanie Garside (Manchester University NHS Foundation Trust, Manchester, UK), Glaxy Gray (Manchester University NHS Foundation Trust, Manchester, UK), Richard Hammonds (Manchester University NHS Foundation Trust, Manchester, UK), Babra Hanif (Manchester University NHS Foundation Trust, Manchester, UK), James Hill (Manchester University NHS Foundation Trust, Manchester, UK), Benjamin Hornung (Manchester University NHS Foundation Trust, Manchester, UK), Anu John (Manchester University NHS Foundation Trust, Manchester, UK), Maya John (Manchester University NHS Foundation Trust, Manchester, UK), Stephen Lee (Manchester University NHS Foundation Trust, Manchester, UK), Jesha Mathews (Manchester University NHS Foundation Trust, Manchester, UK), Pavenjit Nandhra (Manchester University NHS Foundation Trust, Manchester, UK), Mohammed Nazir (Manchester University NHS Foundation Trust, Manchester, UK), Jessica Nichols (Manchester University NHS Foundation Trust, Manchester, UK), Sarah O'Shea (Manchester University NHS Foundation Trust, Manchester, UK), Alice Panes (Manchester University NHS Foundation Trust, Manchester, UK), Laura Perry (Manchester University NHS Foundation Trust, Manchester, UK), Angelique Quistin (Manchester University NHS Foundation Trust, Manchester, UK), Rojy Santosh (Manchester University NHS Foundation Trust, Manchester, UK), Nicholas Stylianides (Manchester University NHS Foundation Trust, Manchester, UK), Jennifer Trezise (Manchester University NHS Foundation Trust, Manchester, UK), Denielle Wilcock (Manchester University NHS Foundation Trust, Manchester, UK), Gian Abbott (Countess of Chester Hospital NHS Foundation Trust, Chester, UK), Paul Evans (Countess of Chester Hospital NHS Foundation Trust, Chester, UK), Claire Gabriel (Countess of Chester Hospital NHS Foundation Trust, Chester, UK), Jenny Grounds (Countess of Chester Hospital NHS Foundation Trust, Chester, UK), Nichola Kearsley (Countess of Chester Hospital NHS Foundation Trust, Chester, UK), Roy Mahapatra (Countess of Chester Hospital NHS Foundation Trust, Chester, UK), Collette Markzu (Countess of Chester Hospital NHS Foundation Trust, Chester, UK), Emmeline Martin (Countess of Chester Hospital NHS Foundation Trust, Chester, UK), Laura Parry (Countess of Chester Hospital NHS Foundation Trust, Chester, UK), Sandra Powell (Countess of Chester Hospital NHS Foundation Trust, Chester, UK), Kunal Rajput (Countess of Chester Hospital NHS Foundation Trust, Chester, UK), Dale Vimalachandran (Countess of Chester Hospital NHS Foundation Trust, Chester, UK), Andrea Young (Countess of Chester Hospital NHS Foundation Trust, Chester, UK), Helen Boros (East Cheshire NHS Trust, Cheshire, UK), Lisa Hardstaff (East Cheshire NHS Trust, Cheshire, UK), Philippa Hill (East Cheshire NHS Trust, Cheshire, UK), Maureen Holland (East Cheshire NHS Trust, Cheshire, UK), Debra Jowle (East Cheshire NHS Trust, Cheshire, UK), Konrad Koss (East Cheshire NHS Trust, Cheshire, UK), Barbara Townley (East Cheshire NHS Trust, Cheshire, UK), Lesley Wilknson (East Cheshire NHS Trust, Cheshire, UK), Hayley Cousins (Hampshire Hospitals NHS Foundation Trust, Hampshire, UK), Barbara King (Hampshire Hospitals NHS Foundation Trust, Hampshire, UK), John Ramage (Hampshire Hospitals NHS Foundation Trust, Hampshire, UK), Amanda Alty (Lancashire Teaching Hospitals NHS Foundation Trust, Lancashire, UK), Paul Barrow (Lancashire Teaching Hospitals NHS Foundation Trust, Lancashire, UK), Alan Beveridge (Lancashire Teaching Hospitals NHS Foundation Trust, Lancashire, UK), Arnab Bhowmick (Lancashire Teaching Hospitals NHS Foundation Trust, Lancashire, UK), Alistair Craig (Lancashire Teaching Hospitals NHS Foundation TrustLancashire, UK), Terri-Louise Cromie (Lancashire Teaching Hospitals NHS Foundation TrustLancashire, UK), Tarek Hany (Lancashire Teaching Hospitals NHS Foundation TrustLancashire, UK), Alka Jadav (Lancashire Teaching Hospitals NHS Foundation Trust, Lancashire, UK), Janet Mills (Lancashire Teaching Hospitals NHS Foundation Trust, Lancashire, UK), Peter Mitchell (Lancashire Teaching Hospitals NHS Foundation Trust, Lancashire, UK), Ed Parkin (Lancashire Teaching Hospitals NHS Foundation Trust, Lancashire, UK), Ioannis Peristerakis (Lancashire Teaching Hospitals NHS Foundation Trust, Lancashire, UK), Sandra Sowden (Lancashire Teaching Hospitals NHS Foundation Trust, Lancashire, UK), Robert Stockwell (Lancashire Teaching Hospitals NHS Foundation Trust, Lancashire, UK), Gagandeep Thind (Lancashire Teaching Hospitals NHS Foundation Trust, Lancashire, UK), Louis Turrel (Lancashire Teaching Hospitals NHS Foundation Trust, Lancashire, UK), Mark Verlander (Lancashire Teaching Hospitals NHS Foundation Trust, Lancashire, UK), Ailsa Watt (Lancashire Teaching Hospitals NHS Foundation Trust, Lancashire, UK), Deborah Weavers (Lancashire Teaching Hospitals NHS Foundation Trust, Lancashire, UK), Alexandra Williams (Lancashire Teaching Hospitals NHS Foundation Trust, Lancashire, UK), Miranda Baum (Leeds Teaching Hospitals NHS Trust, Leeds, UK), Simon Everett (Leeds Teaching Hospitals NHS Trust, Leeds, UK), Vinod Hegade (Leeds Teaching Hospitals NHS Trust, Leeds, UK), Matthew Huggett (Leeds Teaching Hospitals NHS Trust, Leeds, UK), Susan Kelly (Leeds Teaching Hospitals NHS Trust, Leeds, UK), Rebecca King (Leeds Teaching Hospitals NHS Trust, Leeds, UK), Lucy Marshall (Leeds Teaching Hospitals NHS Trust, Leeds, UK), Catherine Moriarty (Leeds Teaching Hospitals NHS Trust, Leeds, UK), Bharat Paranandi (Leeds Teaching Hospitals NHS Trust, Leeds, UK), Mark Priestley (Leeds Teaching Hospitals NHS Trust, Leeds, UK), Rick Saunders (Leeds Teaching Hospitals NHS Trust, Leeds, UK), Holly Speight (Leeds Teaching Hospitals NHS Trust, Leeds, UK), Louise White (Leeds Teaching Hospitals NHS Trust, Leeds, UK), Haidar Alwan-Walker (Manchester University NHS Foundation Trust, Manchester, UK), Helen Ashby (Manchester University NHS Foundation Trust, Manchester, UK), Linda Bailey (Manchester University NHS Foundation Trust, Manchester, UK), Wal Baraza (Manchester University NHS Foundation Trust, Manchester, UK), Molly Bennett (Manchester University NHS Foundation Trust, Manchester, UK), Angela Chrisopoulou (Manchester University NHS Foundation Trust, Manchester, UK), Sarah Duff (Manchester University NHS Foundation Trust, Manchester, UK), Laura Hancock (Manchester University NHS Foundation Trust, Manchester, UK), Zoe Holliday (Manchester University NHS Foundation Trust, Manchester, UK), Javaid Iqbal (Manchester University NHS Foundation Trust, Manchester, UK), Venkata Lekharaju (Manchester University NHS Foundation Trust, Manchester, UK), Fiona McCartin (Manchester University NHS Foundation Trust, Manchester, UK), Gorei Mccavil (Manchester University NHS Foundation Trust, Manchester, UK), Stephen Metcalfe (Manchester University NHS Foundation Trust, Manchester, UK), Heena Mistry (Manchester University NHS Foundation Trust, Manchester, UK), Lindsay Piper (Manchester University NHS Foundation Trust, Manchester, UK), Aswatha Ramesh (Manchester University NHS Foundation Trust, Manchester, UK), Velauthan Rudralingam (Manchester University NHS Foundation Trust, Manchester, UK), Abhiram Sharma (Manchester University NHS Foundation Trust, Manchester, UK), Kathryn Slevin (Manchester University NHS Foundation Trust, Manchester, UK), Karen Telford (Manchester University NHS Foundation Trust, Manchester, UK), Debbie West (Manchester University NHS Foundation Trust, Manchester, UK), Kate Whitehead (Manchester University NHS Foundation Trust, Manchester, UK), Peter Coyne (Newcastle upon Tyne Hospitals NHS Foundation Trust, Newcastle upon Tyne, UK), James Graham (Newcastle upon Tyne Hospitals NHS Foundation Trust, Newcastle upon Tyne, UK), Stephanie Grieveson (Newcastle upon Tyne Hospitals NHS Foundation Trust, Newcastle upon Tyne, UK), Ben Griffiths (Newcastle upon Tyne Hospitals NHS Foundation Trust, Newcastle upon Tyne, UK), Lorna Ingoe (Newcastle upon Tyne Hospitals NHS Foundation Trust, Newcastle upon Tyne, UK), Sam McDonald (Newcastle upon Tyne Hospitals NHS Foundation Trust, Newcastle upon Tyne, UK), Victoria Murtha (Newcastle upon Tyne Hospitals NHS Foundation Trust, Newcastle upon Tyne, UK), Adam Scadeng (Newcastle upon Tyne Hospitals NHS Foundation Trust, Newcastle upon Tyne, UK), Julia Scott (Newcastle upon Tyne Hospitals NHS Foundation Trust, Newcastle upon Tyne, UK), Elaine Stephenson (Newcastle upon Tyne Hospitals NHS Foundation Trust, Newcastle upon Tyne, UK), Vithusa Varnakillasingam (Newcastle upon Tyne Hospitals NHS Foundation Trust, Newcastle upon Tyne, UK), Nelson Wong (Newcastle upon Tyne Hospitals NHS Foundation Trust, Newcastle upon Tyne, UK), Avril Donaldson (NHS Highlnd, Inverness, UK), Kathleen Macleod (NHS Highlnd, Inverness, UK), Andrew Macleod (NHS Highlnd, Inverness, UK), Joanna Matheson (NHS Highlnd, Inverness, UK), Raymond Oliphant (NHS Highlnd, Inverness, UK), Alastair Todd (NHS Highlnd, Inverness, UK), Michael Walker (NHS Highlnd, Inverness, UK), Angus Watson (NHS Highlnd, Inverness, UK), Angie Balfour (NHS Lothian, Edinburgh, UK), Domenyk Brown (NHS Lothian, Edinburgh, UK), John Brush (NHS Lothian, Edinburgh, UK), Stephen Glancy (NHS Lothian, Edinburgh, UK), Sarah Goodbrand (NHS Lothian, Edinburgh, UK), Marion MacRury (NHS Lothian, Edinburgh, UK), Chinnappa Reddy (NHS Lothian, Edinburgh, UK), Doug Speake (NHS Lothian, Edinburgh, UK), Geoff Wogan (NHS Lothian, Edinburgh, UK), Sarah Bevins (North Bristol NHS Trust, Bristol, UK), Kirstie Bradburn (North Bristol NHS Trust, Bristol, UK), Caroline Burt (North Bristol NHS Trust, Bristol, UK), Neil Collin (North Bristol NHS Trust, Bristol, UK), Graham Collin (North Bristol NHS Trust, Bristol, UK), Laura Fox (North Bristol NHS Trust, Bristol, UK), Robert Healey (North Bristol NHS Trust, Bristol, UK), Mitchell Hopes (North Bristol NHS Trust, Bristol, UK), Shinu Jackson (North Bristol NHS Trust, Bristol, UK), Alice Jarvie (North Bristol NHS Trust, Bristol, UK), Regina Kageni (North Bristol NHS Trust, Bristol, UK), Suriya Kirkpatrick (North Bristol NHS Trust, Bristol, UK), Sam Loud (North Bristol NHS Trust, Bristol, UK), Eric Loveday (North Bristol NHS Trust, Bristol, UK), Ann Lyons (North Bristol NHS Trust, Bristol, UK), Kathryn McCarthy (North Bristol NHS Trust, Bristol, UK), Tom McGirr (North Bristol NHS Trust, Bristol, UK), Angus McNair (North Bristol NHS Trust, Bristol, UK), Peter Mezes (North Bristol NHS Trust, Bristol, UK), Emily Perry (North Bristol NHS Trust, Bristol, UK), Anne Pullyblank (North Bristol NHS Trust, Bristol, UK), Sosamma Robin (North Bristol NHS Trust, Bristol, UK), Maricruz Santamaria (North Bristol NHS Trust, Bristol, UK), Alice Smith (North Bristol NHS Trust, Bristol, UK), Andrew Smith (North Bristol NHS Trust, Bristol, UK), Louise Solomon (North Bristol NHS Trust, Bristol, UK), Haytham Sumrien (North Bristol NHS Trust, Bristol, UK), Isileli Tonga (North Bristol NHS Trust, Bristol, UK), Katherine Way (North Bristol NHS Trust, Bristol, UK), David Westwood (North Bristol NHS Trust, Bristol, UK), Guruprasad *P* Aithal (Nottingham University Hospitals NHS Trust, Nottingham, UK), Andrew Baxter (Nottingham University Hospitals NHS Trust, Nottingham, UK), Suzanne Henry (Nottingham University Hospitals NHS Trust, Nottingham, UK), Martin James (Nottingham University Hospitals NHS Trust, Nottingham, UK), Amardeep Khanna (Nottingham University Hospitals NHS Trust, Nottingham, UK), Jodie Newham (Nottingham University Hospitals NHS Trust, Nottingham, UK), Sian Kelly Parkes (Nottingham University Hospitals NHS Trust, Nottingham, UK), Stephen Ryder (Nottingham University Hospitals NHS Trust, Nottingham, UK), Ioannis Varmpompitis (Nottingham University Hospitals NHS Trust, Nottingham, UK), Suresh Vasan Venkatachalapathy (Nottingham University Hospitals NHS Trust, Nottingham, UK), Samantha Warburton (Nottingham University Hospitals NHS Trust, Nottingham, UK), Sarah Askew (Royal Cornwall Hospitals NHS Trust, Cornwall, UK), Kerry Atkinson (Royal Cornwall Hospitals NHS Trust, Cornwall, UK), Madalina Chifu (Royal Cornwall Hospitals NHS Trust, Cornwall, UK), Candy Coombe (Royal Cornwall Hospitals NHS Trust, Cornwall, UK), Sophia Eloi (Royal Cornwall Hospitals NHS Trust, Cornwall, UK), Clare Ferris (Royal Cornwall Hospitals NHS Trust, Cornwall, UK), Elizabeth Firth (Royal Cornwall Hospitals NHS Trust, Cornwall, UK), Caroline Goddard (Royal Cornwall Hospitals NHS Trust, Cornwall, UK), Anne Griffiths (Royal Cornwall Hospitals NHS Trust, Cornwall, UK), John Hancock (Royal Cornwall Hospitals NHS Trust, Cornwall, UK), Angela Irving (Royal Cornwall Hospitals NHS Trust, Cornwall, UK), Kirsty Maclean (Royal Cornwall Hospitals NHS Trust, Cornwall, UK), John Madine (Royal Cornwall Hospitals NHS Trust, Cornwall, UK), Corrine Penhaligon (Royal Cornwall Hospitals NHS Trust, Cornwall, UK), Catherine Pentescost (Royal Cornwall Hospitals NHS Trust, Cornwall, UK), Kirsty Prout (Royal Cornwall Hospitals NHS Trust, Cornwall, UK), Rebecca Rogers (Royal Cornwall Hospitals NHS Trust, Cornwall, UK), Rebecca Sargent (Royal Cornwall Hospitals NHS Trust, Cornwall, UK), Anita Steele (Royal Cornwall Hospitals NHS Trust, Cornwall, UK), Felicity Verma (Royal Cornwall Hospitals NHS Trust, Cornwall, UK), Michael Agyemang (Sheffield Teaching Hospitals NHS Foundation Trust, Sheffield, UK), Sarah Bird(Sheffield Teaching Hospitals NHS Foundation Trust, Sheffield, UK), Steven Brown (Sheffield Teaching Hospitals NHS Foundation Trust, Sheffield, UK), Holly Caborn (Sheffield Teaching Hospitals NHS Foundation Trust, Sheffield, UK), Joyce Fofie (Sheffield Teaching Hospitals NHS Foundation Trust, Sheffield, UK), James Hampton (Sheffield Teaching Hospitals NHS Foundation Trust, Sheffield, UK), Faith Kibutu (Sheffield Teaching Hospitals NHS Foundation Trust, Sheffield, UK), Fred Lee (Sheffield Teaching Hospitals NHS Foundation Trust, Sheffield, UK), Cecilia Mason (Sheffield Teaching Hospitals NHS Foundation Trust, Sheffield, UK), Angeline Mbuyisa (Sheffield Teaching Hospitals NHS Foundation Trust, Sheffield, UK), Helen Newell (Sheffield Teaching Hospitals NHS Foundation Trust, Sheffield, UK), Viktoria Cripps (Somerset NHS Foundation Trust, Somerset, UK), Thomas Edwards (Somerset NHS Foundation Trust, Somerset, UK), Nicky Forsyth (Somerset NHS Foundation Trust, Somerset, UK), Louise Hunt (Somerset NHS Foundation Trust, Somerset, UK), Andrew Lowe (Somerset NHS Foundation Trust, Somerset, UK), Paul Mackey (Somerset NHS Foundation Trust, Somerset, UK), Rudi Matull (Somerset NHS Foundation Trust, Somerset, UK), Alison Moss (Somerset NHS Foundation Trust, Somerset, UK), Corinne Pawley (Somerset NHS Foundation Trust, Somerset, UK), Tamlyn Russell (Somerset NHS Foundation Trust, Somerset, UK), Maria Salter (Somerset NHS Foundation Trust, Somerset, UK), Charmaine Shovelton (Somerset NHS Foundation Trust, Somerset, UK), Edward Smyth (Somerset NHS Foundation Trust, Somerset, UK), Angela Berry (South Eastern Health and Social Care Trust, Dundonald, UK), Nicola Broome (South Eastern Health and Social Care Trust, Dundonald, UK), Grant Caddy (South Eastern Health and Social Care Trust, Dundonald, UK), John Eccles (South Eastern Health and Social Care Trust, Dundonald, UK), Jennifer Foreman (South Eastern Health and Social Care Trust, Dundonald, UK), Tony Tham (South Eastern Health and Social Care Trust, Dundonald, UK), Alex Usher-Rea (South Eastern Health and Social Care Trust, Dundonald, UK), Andrew Wray (South Eastern Health and Social Care Trust, Dundonald, UK), Gail Young (South Eastern Health and Social Care Trust, Dundonald, UK), Harry Bond (The Christie NHS Foundation Trust, Manchester, UK), Theresa Taylor Emberton (The Christie NHS Foundation Trust, Manchester, UK), Hans-Ulrich Laasch (The Christie NHS Foundation Trust, Manchester, UK), Sue Lane (Nee Fenton) (The Christie NHS Foundation Trust, Manchester, UK), Damian Mullan (The Christie NHS Foundation Trust, Manchester, UK), Lyne Robertson (The Christie NHS Foundation Trust, Manchester, UK), Maria RoyoGamara (The Christie NHS Foundation Trust, Manchester, UK), Marie Green (The Royal Wolverhampton NHS Trust, Wolverhampton, UK), Brian McKaig (The Royal Wolverhampton NHS Trust, Wolverhampton, UK), Shyam Menon (The Royal Wolverhampton NHS Trust, Wolverhampton, UK), Rajinder Nayyar (The Royal Wolverhampton NHS Trust, Wolverhampton, UK), Julie Roberts (The Royal Wolverhampton NHS Trust, Wolverhampton, UK), Helen Steed (The Royal Wolverhampton NHS Trust, Wolverhampton, UK), Andrew Veitch (The Royal Wolverhampton NHS Trust, Wolverhampton, UK), Sarah Addison (University Hospitals Birmingham NHS Foundation Trust, Birmingham, UK), Shazad Ashraf (University Hospitals Birmingham NHS Foundation Trust, Birmingham, UK), Simon Bach (University Hospitals Birmingham NHS Foundation Trust, Birmingham, UK), Anil Bagul (University Hospitals Birmingham NHS Foundation Trust, Birmingham, UK), Elizabeth Bailey (University Hospitals Birmingham NHS Foundation Trust, Birmingham, UK), Andrew Beggs (University Hospitals Birmingham NHS Foundation Trust, Birmingham, UK), Colm Forde (University Hospitals Birmingham NHS Foundation Trust, Birmingham, UK), Sharon Garner (University Hospitals Birmingham NHS Foundation Trust, Birmingham, UK), Manijeh Ghods (University Hospitals Birmingham NHS Foundation Trust, Birmingham, UK), Andrew McDarby (University Hospitals Birmingham NHS Foundation Trust, Birmingham, UK), Claire McNeill (University Hospitals Birmingham NHS Foundation Trust, Birmingham, UK), Dion Morton (University Hospitals Birmingham NHS Foundation Trust, Birmingham, UK), Dimitri Nepogodiev (University Hospitals Birmingham NHS Foundation Trust, Birmingham, UK), Arvind Pallan (University Hospitals Birmingham NHS Foundation Trust, Birmingham, UK), Tom Pinkney (University Hospitals Birmingham NHS Foundation Trust, Birmingham, UK), Jonathan Richardson (University Hospitals Birmingham NHS Foundation Trust, Birmingham, UK), Nigel Suggett (University Hospitals Birmingham NHS Foundation Trust, Birmingham, UK), Sharan Wadhwani (University Hospitals Birmingham NHS Foundation Trust, Birmingham, UK), Stephan Ward (University Hospitals Birmingham NHS Foundation Trust, Birmingham, UK), Arlo Whithouse (University Hospitals Birmingham NHS Foundation Trust, Birmingham, UK), Deborah Wright (University Hospitals Birmingham NHS Foundation Trust, Birmingham, UK), Hasan Al Chalabi (University Hospitals of Derby and Burton NHS Foundation Trust, Derby, UK), Ashish Bhalla (University Hospitals of Derby and Burton NHS Foundation Trust, Derby, UK), Jo Chmiel (University Hospitals of Derby and Burton NHS Foundation Trust, Derby, UK), Julie Edmonds (University Hospitals of Derby and Burton NHS Foundation Trust, Derby, UK), Jodie Fitzgerald (University Hospitals of Derby and Burton NHS Foundation Trust, Derby, UK), Nicole Isitt (University Hospitals of Derby and Burton NHS Foundation Trust, Derby, UK), Jonathan Lund (University Hospitals of Derby and Burton NHS Foundation Trust, Derby, UK), Nicole Mckee (University Hospitals of Derby and Burton NHS Foundation Trust, Derby, UK), Joely Morgan (University Hospitals of Derby and Burton NHS Foundation Trust, Derby, UK), Elizabeth Nadin (University Hospitals of Derby and Burton NHS Foundation Trust, Derby, UK), Ellie Piggott (University Hospitals of Derby and Burton NHS Foundation Trust, Derby, UK), Rajeev Singh (University Hospitals of Derby and Burton NHS Foundation Trust, Derby, UK), Katherine Smith (University Hospitals of Derby and Burton NHS Foundation Trust, Derby, UK), William Speake (University Hospitals of Derby and Burton NHS Foundation Trust, Derby, UK), Peter Thurley (University Hospitals of Derby and Burton NHS Foundation Trust, Derby, UK), Samson Tou (University Hospitals of Derby and Burton NHS Foundation Trust, Derby, UK), Christ Worth (University Hospitals of Derby and Burton NHS Foundation Trust, Derby, UK), Jill Cooke (University Hospitals of Leicester NHS Trust, Leicester, UK), Rachel Plummer (University Hospitals of Leicester NHS Trust, Leicester, UK), Baljit Singh (University Hospitals of Leicester NHS Trust, Leicester, UK), Ratan Verma (University Hospitals of Leicester NHS Trust, Leicester, UK), Ndkeita Barnett (University Hospitals of North Midlands NHS Trust, Stoke-on-Trent, UK), Adrian Butler (University Hospitals of North Midlands NHS Trust, Stoke-on-Trent, UK), Susan Gallagher (University Hospitals of North Midlands NHS Trust, Stoke-on-Trent, UK), Amanda Hall (University Hospitals of North Midlands NHS Trust, Stoke-on-Trent, UK), Kar Wai Lau (University Hospitals of North Midlands NHS Trust, Stoke-on-Trent, UK), Mia Marsden (University Hospitals of North Midlands NHS Trust, Stoke-on-Trent, UK), Michael Martin (University Hospitals of North Midlands NHS Trust, Stoke-on-Trent, UK), Katrina Parkinson (University Hospitals of North Midlands NHS Trust, Stoke-on-Trent, UK), Rochelle Rhodes (University Hospitals of North Midlands NHS Trust, Stoke-on-Trent, UK), Alison Tilley (York and Scarborough Teaching Hospitals NHS Foundation Trust, York, UK) Laura Barman, Kerry Elliot (York and Scarborough Teaching Hospitals NHS Foundation Trust, York, UK), Janine Mallinson (York and Scarborough Teaching Hospitals NHS Foundation Trust, York, UK), Tania Neale (York and Scarborough Teaching Hospitals NHS Foundation Trust, York, UK), Ian Renwick (York and Scarborough Teaching Hospitals NHS Foundation Trust, York, UK), Jacqui Smith (York and Scarborough Teaching Hospitals NHS Foundation Trust, York, UK), Alison Turnbull (York and Scarborough Teaching Hospitals NHS Foundation Trust, York, UK).

## Supplementary Material

znaf117_Supplementary_Data

## Data Availability

CReST2 adopts a controlled access data sharing policy. All data requests should be submitted to the corresponding author for consideration. Access to anonymized data may be granted after review by the BCTU data sharing committee.
