# Supplementary material for: Covered versus uncovered endoluminal stenting in the acute management of obstructing colorectal cancer in the palliative setting: randomized clinical trial (CReST2)
Source: Br J Surg. 2025 Sep 17;112(9):znaf117. doi: 10.1093/bjs/znaf117 (PMC12448851; doi:10.1093/bjs/znaf117)
Supplement: znaf117_Supplementary_Data [file znaf117_supplementary_data.docx]

**Title: ColoRectal Stenting Trial 2 (CReST2)- Covered vs uncovered endoluminal stenting in the acute management of obstructing colorectal cancer in the palliative setting: randomised clinical trial**

CReST2 Collaborative Group

**Corresponding author.** James Hill, Division of Surgery, Department of General Surgery, Manchester Royal Infirmary, Oxford Road, Manchester M13 9WL, UK (e-mail: James.hill1101@gmail.com)

**Supplementary Materials - Index**

| **Supplementary Methods** |  |
| --- | --- |
| Patient Pathway | *pag. 2* |
| **Supplementary Results** |  |
| Baseline characteristics for modified ITT analysis dataset | *pag. 3* |
| Stent insertion procedure details | *pag. 4* |
| Subgroup analyses of quality of life primary outcome | *pag. 6* |
| Raw QLQ-C30 global health score data by time-point | *pag. 9* |
| Subgroup analyses of stent patency primary outcome | *pag. 10* |
| Sensitivity analyses | *pag. 13* |
| QLQ-CR29 quality of life questionnaire | *pag. 16* |
| **Supplementary Appendixes** |  |
| Collaborators List | *pag. 39* |

**Supplementary Methods**

**Patient Pathway**


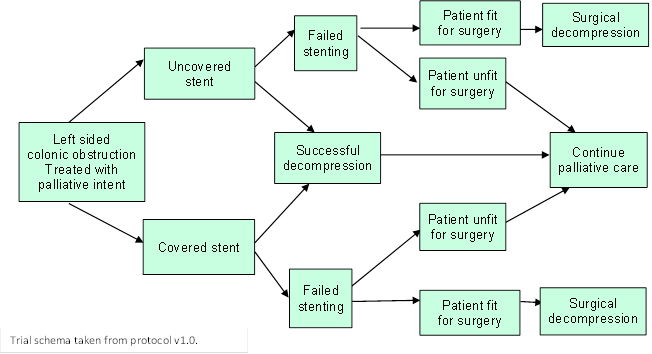


**Supplementary Results**

**Baseline characteristics for modified ITT analysis dataset**

|  |  | | | **Covered**  **stent**  **n=161** | **Uncovered**  **stent**  **n=166** | **Overall**  **n=327** |
| --- | --- | --- | --- | --- | --- | --- |
| **Minimisation variables** | | | | | | |
| Age (years) | <=70 | | | 64 (39.8%) | 57 (34.3%) | 121 (37.0%) |
|  | >70 | | | 97 (60.2%) | 109 (65.7%) | 206 (63.0%) |
| WHO performance status | 0 – fully active | | | 53 (32.9%) | 47 (28.3%) | 100 (30.6%) |
|  | 1 – mobile all day | | | 60 (37.3%) | 70 (42.2%) | 130 (39.8%) |
|  | 2 – in bed <50% | | | 32 (19.9%) | 35 (21.1%) | 67 (20.5%) |
|  | 3 – in bed >50% | | | 14 (8.7%) | 12 (7.2%) | 26 (8.0%) |
|  | 4 – bedridden | | | 2 (1.2%) | 2 (1.2%) | 4 (1.2%) |
| Primary tumour site | | Ascending colon | | 3 (1.9%) | 2 (1.2%) | 5 (1.5%) |
|  |  | Hepatic flexure | | 2 (1.2%) | 6 (3.6%) | 8 (2.4%) |
|  |  | Transverse colon | | 15 (9.3%) | 14 (8.4%) | 29 (8.9%) |
|  |  | Splenic flexure | | 12 (7.5%) | 12 (7.2%) | 24 (7.3%) |
|  |  | Descending colon | | 21 (13.0%) | 23 (13.9%) | 44 (13.5%) |
|  |  | Sigmoid | | 75 (46.6%) | 81 (48.8%) | 156 (47.7%) |
|  |  | Rectosigmoid | | 24 (14.9%) | 23 (13.9%) | 47 (14.4%) |
|  |  | Rectum (proximal) | | 9 (5.6%) | 5 (3.0%) | 14 (4.3%) |
| Indication for palliation | Unresectable local disease | | | 42 (26.1%) | 44 (26.5%) | 86 (26.3%) |
|  | Unresectable metastatic disease | | | 80 (49.7%) | 79 (47.6%) | 159 (48.6%) |
|  | Considered unfit for surgery | | | 39 (24.2%) | 43 (25.9%) | 82 (25.1%) |
| **Demographic and other baseline variables** | | | | | | |
| Age (years) | Mean (SD) | | | 72.1 (15.4) | 74.6 (13.2) | 73.4 (14.3) |
|  | Range | | | 27.0 - 98.0 | 32.0 - 98.0 | 27.0 - 98.0 |
|  | Missing | | | 0 | 0 | 0 |
| Sex ratio (M : F) | | | | 110 : 51 | 101 : 65 | 211 : 116 |
| Method of diagnosis* | | | Colonoscopy | 57 (35.4%) | 70 (42.2%) | 127 (38.8%) |
|  |  |  | CT scan | 151 (93.8%) | 153 (92.2%) | 304 (93.0%) |
|  |  |  | Flexible sigmoidoscopy | 52 (32.3%) | 50 (30.1%) | 102 (31.2%) |
|  |  |  | MRI | 14 (8.7%) | 14 (8.4%) | 28 (8.6%) |
| Metastases diagnosed | No | | | 46 (28.6%) | 45 (27.1%) | 91 (27.8%) |
|  | Yes | | | 115 (71.4%) | 121 (72.9%) | 236 (72.2%) |
|  | Missing | | | 0 (0.0%) | 0 (0.0%) | 0 (0.0%) |

*Note that more than one method of diagnosis can be specified; therefore, the sum of the numbers presented may exceed the total number of randomised patients on the trial.

**Stent insertion procedure details**

|  | **Covered**  **stent**  **(n=188)** | **Uncovered**  **Stent**  **(n=186)** |
| --- | --- | --- |
|  |  |  |
| **Was stent insertion technically successful?** |  |  |
| **Yes** | **161 (85.6%)** | **166 (89.2%)** |
| **No / not stented / form missing** | **27 (14.4%)** | **20 (10.8%)** |
|  |  |  |
| **WHERE STENT INSERTION WAS NOT TECHNICALLY SUCCESSFUL** | | |
|  | **N=27** | **N=20** |
| **Drugs administered*** |  |  |
| Midazolam | 21 (78%) | 14 (70%) |
| Fentanyl | 21 (78%) | 12 (60%) |
| Flumenazil | - | - |
| Other | 6 (22%) | 1 (5%) |
| Not on any drugs | 2 (7%) | 4 (20%) |
|  |  |  |
| **Reason why stent insertion was not technically successful*** |  |  |
| Unable to reach stricture | 2 (7%) | 1 (5%) |
| Unable to pass guidewire | 16 (59%) | 10 (50%) |
| Failure to deploy | 0 (0%) | 2 (10%) |
| Stent mis-placement | - | - |
| Other | 8 (30%) | 8 (40%) |
| No any reason given | 1 (4%) | 2 (10%) |
|  |  |  |
| **Subsequent treatment** |  |  |
| Subsequent successful stenting alone | 3 | 2 |
| Surgery alone | 15 | 9 |
| Stenting and surgery | 0 | 1 |
| No further treatment | 9 | 8 |
|  |  |  |
|  |  |  |
| **WHERE STENT INSERTION WAS TECHNICALLY SUCCESSFUL** | | |
|  | **N=161** | **N=166** |
| **Guidance method** |  |  |
| Endoscopic only | **-** | **-** |
| Fluoroscopic only | 6 (3.7%) | 3 (1.8%) |
| Endoscopic and fluoroscopic | 151 (93.8%) | 156 (94.0%) |
| Missing | 4 (2.5%) | 7 (4.2%) |
| **Stent site** |  |  |
| Ascending colon | 2 (1.2%) | 0 (0.0%) |
| Hepatic flexure | 3 (1.9%) | 6 (3.6%) |
| Transverse colon | 17 (10.6%) | 13 (7.8%) |
| Splenic flexure | 13 (8.1%) | 13 (7.8%) |
| Descending colon | 20 (12.4%) | 26 (15.7%) |
| Sigmoid | 68 (42.2%) | 74 (44.6%) |
| Rectosigmoid | 26 (16.2%) | 26 (15.7%) |
| Rectum (proximal) | 12 (7.5%) | 7 (4.2%) |
| Missing | 0 (0.0%) | 1 (0.6%) |
|  |  |  |
| **Duration of procedure (minutes)** |  |  |
| Mean (s.d.) | 33.4 (22.1) | 35.4 (22.9) |
| Median (IQR) | 26 (20, 40) | 30 (20, 45) |
| Min, max | 5, 120 | 10, 180 |
| Missing | 6 | 7 |
|  |  |  |
| **Stent expansion (%)** |  |  |
| Mean (s.d.) | 40.7 (22.0) | 44.9 (21.5) |
| Median (IQR) | 33 (25, 50) | 45.5 (25, 60) |
| Min, max | 10, 100 | 10, 100 |
| Missing | 6 | 8 |
| **Drugs administered*** |  |  |
| Midazolam | 121 (75.2%) | 141 (84.9%) |
| Fentanyl | 101 (62.7%) | 119 (71.7%) |
| Flumenazil | 7 (4.4%) | 2 (1.2%) |
| Other | 22 (13.7%) | 30 (18.1%) |
| Not on any drugs | 22 (13.7%) | 9 (5.4%) |

*Note that more than one option can be selected; therefore, the sum of the numbers presented may exceed the total number of randomised patients.

**Subgroup analyses of quality of life primary outcome**

**Age**

|  | | **Covered stent**  **Mean (SD), n** | **Uncovered stent**  **Mean (SD), n** | **Interaction p-value** | **Mean difference^1^**  **(97.5% CI)** |
| --- | --- | --- | --- | --- | --- |
| Age (years) | <=70 | 59.6 (25.6), 40 | 59.7 (22.8), 30 | 0.684 | -0.75 (-13.95, 12.44) |
|  | >70 | 50.7 (22.4), 64 | 48.6 (25.8), 82 |  | 2.14 (-6.93, 11.22) |

^1^Mean difference (covered - uncovered) calculated using linear regression model, adjusting for baseline global health score, age, WHO performance status, tumour site, indication for palliation and treatment by age interaction, where tumour site variable was collapsed into right colon (ascending colon, hepatic flexure, transverse colon, splenic flexure) and left colon/rectum (descending colon, sigmoid, rectosigmoid, rectum (proximal)). Higher mean scores indicate higher quality of life. A positive difference favours the covered group. Missing values for global health score at month 3 have been imputed using the corresponding score at 30 days where available. The analysis was conducted on all randomised patients who had been successfully stented and had QLQ-C30 scores at baseline and at either 30 days or 3 months post randomisation.

**Tumour site**

|  | | **Covered stent**  **Mean (SD), n** | **Uncovered stent**  **Mean (SD), n** | **Interaction p-value** | **Mean difference^1^**  **(97.5% CI)** |
| --- | --- | --- | --- | --- | --- |
| Tumour site | Right colon | 50.4 (22.5), 23 | 48.0 (27.2), 25 | 0.704 | 4.03 (-12.09, 20.15) |
|  | Left colon/rectum | 55.1 (24.4), 81 | 52.6 (24.9), 87 |  | 0.93 (-7.64, 9.50) |

^1^Mean difference (covered - uncovered) calculated using linear regression model, adjusting for baseline global health score, age, WHO performance status, tumour site, indication for palliation and treatment by tumour site interaction, where tumour site variable was collapsed into right colon (ascending colon, hepatic flexure, transverse colon, splenic flexure) and left colon/rectum (descending colon, sigmoid, rectosigmoid, rectum (proximal)). Higher mean scores indicate higher quality of life. A positive difference favours the covered group. Missing values for global health score at month 3 have been imputed using the corresponding score at 30 days where available. The analysis was conducted on all randomised patients who had been successfully stented and had QLQ-C30 scores at baseline and at either 30 days or 3 months post randomisation.

**WHO performance status**

|  | | **Covered stent**  **Mean (SD), n** | **Uncovered stent**  **Mean (SD), n** | **Interaction p-value** | **Mean difference^1^**  **(97.5% CI)** |
| --- | --- | --- | --- | --- | --- |
| WHO status | Fully active | 60.2 (25.0), 36 | 58.3 (24.0), 35 | 0.802 | 0.00 (-13.25, 13.25) |
|  | Mobile all day | 54.3 (21.8), 41 | 49.7 (22.6), 50 |  | 4.87 (-6.77, 16.51) |
|  | In bed <50% | 45.2 (22.6), 19 | 48.7 (32.1), 19 |  | -5.69 (-23.73, 12.34) |
|  | In bed ≥50% | 46.4 (30.4), 7 | 38.1 (29.6), 7 |  | 8.75 (-20.68, 38.19) |
|  | Bedridden | 50.0 (-), 1 | 58.3 (-), 1 |  | -4.19 (-82.52, 74.13) |

^1^Mean difference (covered - uncovered) calculated using linear regression model, adjusting for baseline global health score, age, WHO performance status, tumour site, indication for palliation and treatment by WHO performance status interaction, where tumour site variable was collapsed into right colon (ascending colon, hepatic flexure, transverse colon, splenic flexure) and left colon/rectum (descending colon, sigmoid, rectosigmoid, rectum (proximal)). Higher mean scores indicate higher quality of life. A positive difference favours the covered group. Missing values for global health score at month 3 have been imputed using the corresponding score at 30 days where available. The analysis was conducted on all randomised patients who had been successfully stented and had QLQ-C30 scores at baseline and at either 30 days or 3 months post randomisation.

**Indication for palliation**

|  | | **Covered stent**  **Mean (SD), n** | **Uncovered stent**  **Mean (SD), n** | **Interaction p-value** | **Mean difference^1^**  **(97.5% CI)** |
| --- | --- | --- | --- | --- | --- |
| Indication for palliation | Unresectable local | 50.9 (19.9), 28 | 52.5 (24.1), 30 | 0.480 | -4.86 (-19.68, 9.96) |
|  | Unresectable metastatic | 57.7 (26.7), 50 | 53.0 (27.5), 50 |  | 5.05 (-5.98, 16.07) |
|  | Unfit for surgery | 50.6 (22.1), 26 | 48.4 (23.6), 32 |  | 2.13 (-12.56, 16.82) |

^1^Mean difference (covered - uncovered) calculated using linear regression model, adjusting for baseline global health score, age, WHO performance status, tumour site, indication for palliation and treatment by indication for palliation interaction, where tumour site variable was collapsed into right colon (ascending colon, hepatic flexure, transverse colon, splenic flexure) and left colon/rectum (descending colon, sigmoid, rectosigmoid, rectum (proximal)). Higher mean scores indicate higher quality of life. A positive difference favours the covered group. Missing values for global health score at month 3 have been imputed using the corresponding score at 30 days where available. The analysis was conducted on all randomised patients who had been successfully stented and had QLQ-C30 scores at baseline and at either 30 days or 3 months post randomisation.

**Raw QLQ-C30 global health score data by time-point**

| **QLQ-C30 global health score^*^** | **covered stent** | | **uncovered stent** | |
| --- | --- | --- | --- | --- |
|  | **Mean (SD)** | **N** | **Mean (SD)** | **N** |
| Baseline | 48.22 (23.84) | 150 | 43.70 (26.43) | 156 |
| 30 days | 54.38 (22.28) | 97 | 55.13 (23.28) | 112 |
| 3 months | 56.62 (23.55) | 78 | 51.24 (24.00) | 74 |
| 6 months | 61.70 (21.76) | 57 | 51.53 (25.79) | 60 |
| 12 months | 58.78 (25.38) | 37 | 55.65 (23.21) | 31 |
| 18 months | 64.91 (18.96) | 19 | 62.04 (21.43) | 18 |
| 24 months | 54.17 (20.41) | 8 | 51.67 (19.56) | 10 |
| *Including all available QLQ-C30 global health score at each time point for patients with a successful first stent insertion. Missing values for global health score at month 3 have NOT been imputed using the corresponding score at 30 days. Higher mean scores indicate higher quality of life. | | | | |

**Subgroup analyses of stent patency primary outcome**

**Age**

|  | | **Stent failure** | **covered stent**  **(n=161)** | **uncovered stent (control)**  **(n=166)** | **Interaction p-value** | **Adjusted HR^1^**  **(97.5% CI)** |
| --- | --- | --- | --- | --- | --- | --- |
| Age (years) | <=70 | No | 43 (68.3%) | 35 (67.3%) | 0.028 | 0.85 (0.40, 1.80) |
|  |  | Yes | 20 (31.8%) | 17 (32.7%) |  |  |
|  | >70 | No | 74 (75.5%) | 101 (88.6%) |  | 2.49 (1.13, 5.47) |
|  |  | Yes | 24 (24.5%) | 13 (11.4%) |  |  |

^1^Cox proportional hazards model, adjusting for age, WHO performance status, tumour site, indication for palliation and treatment by age interaction, where tumour site variable was collapsed into right colon (ascending colon, hepatic flexure, transverse colon, splenic flexure) and left colon/rectum (descending colon, sigmoid, rectosigmoid, rectum (proximal)). A hazard ratio < 1 favours the covered group. The analysis was conducted on all randomised patients who had been successfully stented.

**WHO performance status**

|  | | **Stent failure** | **covered stent**  **(n=161)** | **uncovered stent (control)**  **(n=166)** | **Interaction p-value** | **Adjusted HR^1^**  **(97.5% CI)** |
| --- | --- | --- | --- | --- | --- | --- |
| WHO performance status | Fully active | No | 35 (66.0%) | 34 (72.3%) | 0.335^2^ | 1.01 (0.44, 2.31) |
|  |  | Yes | 18 (34.0%) | 13 (27.7%) |  |  |
|  | Mobile all day | No | 47 (78.3%) | 58 (82.9%) |  | 1.38 (0.56, 3.39) |
|  |  | Yes | 13 (21.7%) | 12 (17.1%) |  |  |
|  | In bed <50% | No | 24 (75.0%) | 33 (94.3%) |  | 4.83 (0.81, 28.79) |
|  |  | Yes | 8 (25.0%) | 2 (5.7%) |  |  |
|  | In bed ≥50% | No | 11 (78.6%) | 9 (75.0%) |  | 0.91 (0.15, 5.71) |
|  |  | Yes | 3 (21.4%) | 3 (25.0%) |  |  |
|  | Bedridden | No | 0 (0.0%) | 2 (100.0%) |  | - |
|  |  | Yes | 2 (100.0%) | 0 (0.0%) |  |  |

^1^Cox proportional hazards model, adjusting for age, WHO performance status, tumour site, indication for palliation and treatment by WHO performance status interaction, where tumour site variable was collapsed into right colon (ascending colon, hepatic flexure, transverse colon, splenic flexure) and left colon/rectum (descending colon, sigmoid, rectosigmoid, rectum (proximal)). A hazard ratio < 1 favours the covered group. The analysis was conducted on all randomised patients who had been successfully stented.

^2^the p-value was produced based on testparm command in Stata, with constraint for subgroup “Bedridden” dropped which only contains 4 participants in total.

**Tumour site**

|  | | **Stent failure** | **covered stent**  **(n=161)** | **uncovered stent (control)**  **(n=166)** | **Interaction p-value** | **Adjusted HR^1^**  **(97.5% CI)** |
| --- | --- | --- | --- | --- | --- | --- |
| Tumour site | Right colon | No | 29 (90.6%) | 31 (91.2%) | 0.670 | 1.06 (0.17, 6.69) |
|  |  | Yes | 3 (9.4%) | 3 (8.8%) |  |  |
|  | Left colon/rectum | No | 88 (68.2%) | 105 (79.6%) |  | 1.53 (0.87, 2.71) |
|  |  | Yes | 41 (31.8%) | 27 (20.5%) |  |  |

^1^Cox proportional hazards model, adjusting for age, WHO performance status, tumour site, indication for palliation and treatment by tumour site interaction, where tumour site variable was collapsed into right colon (ascending colon, hepatic flexure, transverse colon, splenic flexure) and left colon/rectum (descending colon, sigmoid, rectosigmoid, rectum (proximal)). A hazard ratio < 1 favours the covered group. The analysis was conducted on all randomised patients who had been successfully stented.

**Indication for palliation**

|  | | **Stent failure** | **covered stent**  **(n=161)** | **uncovered stent (control)**  **(n=166)** | **Interaction p-value** | **Adjusted HR^1^**  **(97.5% CI)** |
| --- | --- | --- | --- | --- | --- | --- |
| Indication for palliation | Unresectable local | No | 28 (66.7%) | 34 (77.3%) | 0.610 | 1.22 (0.46, 3.22) |
|  |  | Yes | 14 (33.3%) | 10 (22.7%) |  |  |
|  | Unresectable metastatic | No | 60 (75.0%) | 65 (82.3%) |  | 1.35 (0.62, 2.97) |
|  |  | Yes | 20 (25.0%) | 14 (17.7%) |  |  |
|  | Unfit for surgery | No | 29 (74.4%) | 37 (86.1%) |  | 2.31 (0.72, 7.37) |
|  |  | Yes | 10 (25.6%) | 6 (14.0%) |  |  |

^1^Cox proportional hazards model, adjusting for age, WHO performance status, tumour site, indication for palliation and treatment by indication for palliation interaction, where tumour site variable was collapsed into right colon (ascending colon, hepatic flexure, transverse colon, splenic flexure) and left colon/rectum (descending colon, sigmoid, rectosigmoid, rectum (proximal)). A hazard ratio < 1 favours the covered group. The analysis was conducted on all randomised patients who had been successfully stented.

**Sensitivity analyses**

### Quality of Life (entire cohort)

The primary quality of life analysis only considered those participants who had a successful stent insertion procedure. This sensitivity analysis includes all randomised participants (excluding the 3 complete withdrawals) regardless of the success of their stenting procedure. This sensitivity analysis has been performed to test the robustness of the primary quality of life analysis.

|  | **covered stent** | **uncovered stent**  **(control)** | **Estimate (97.5%CI)** ^1^ | **P-value** |
| --- | --- | --- | --- | --- |
| Number in analysis dataset | 188 | 186 |  |  |
| Those in analysis dataset who have a baseline global health score | 172 | 174 |  |  |
| Those in analysis dataset with baseline global health score, AND at either 30 days or at 3 months post randomisation | 117 | 120 |  |  |
| *3 month score available* | *79* | *77* |  |  |
| *30 day score carried forward* | *38* | *43* |  |  |
|  |  |  |  |  |
| Global health score at baseline | 48.6 (23.8) | 46.7 (26.2) |  |  |
| Global health score at month 3 (Mean (SD)) | 53.9 (24.4) | 51.2 (25.2) | 2.36  (-4.80, 9.52) | 0.458 ^2^ |
|  |  |  | 1.78  (-5.58, 9.14) | 0.586 ^3^ |
|  |  |  | 1.76  (-5.46, 8.98) | 0.583 ^4^ |

^1^ Higher mean scores indicate higher quality of life. A positive difference favours the covered group. Missing values for global health score at month 3 have been imputed using the corresponding score at 30 days where available.

^2^ Mean difference (covered - uncovered) calculated using a linear regression model adjusted for baseline global health score.

^3^ Mean difference (covered - uncovered) calculated using a linear regression model, adjusting for baseline global health score, age, WHO performance status, tumour site, and indication for palliation.

^4^ As in (2) but tumour site variable collapsed into right colon (ascending colon, hepatic flexure, transverse colon, splenic flexure) and left colon/rectum (descending colon, sigmoid, rectosigmoid, rectum (proximal)).

### Quality of Life (participants who were adherent to their randomised allocation)

The primary quality of life analysis considered all participants regardless of their adherence to their randomised allocation. This sensitivity analysis restricts the analysis dataset to those participants with successful stent insertions who were adherent to their randomised allocation. This sensitivity analysis has been performed to test the robustness of the primary quality of life analysis.

|  | **covered stent** | **uncovered stent**  **(control)** | **Estimate (97.5%CI)** ^1^ | **P-value** |
| --- | --- | --- | --- | --- |
| Number in analysis dataset | 161 | 166 |  |  |
| Those who are adherent | 153 | 161 |  |  |
| Those adherent in analysis dataset who have a baseline global health score | 142 | 151 |  |  |
| Those adherent in analysis dataset with baseline global health score, AND at either 30 days or at 3 months post randomisation | 99 | 107 |  |  |
| *3 month score available* | *68* | *68* |  |  |
| *30 day score carried forward* | *31* | *39* |  |  |
|  |  |  |  |  |
| Global health score at baseline | 49.4 (23.5) | 47.3 (26.5) |  |  |
| Global health score at month 3 (Mean (SD)) | 54.8 (24.2) | 52.0 (25.3) | 2.24  (-5.33, 9.82) | 0.504 ^2^ |
|  |  |  | 1.93  (-5.85, 9.71) | 0.576 ^3^ |
|  |  |  | 1.91  (-5.70, 9.53) | 0.571 ^4^ |

^1^ Higher mean scores indicate higher quality of life. A positive difference favours the covered group. Missing values for global health score at month 3 have been imputed using the corresponding score at 30 days where available.

^2^ Mean difference (covered - uncovered) calculated using a linear regression model adjusted for baseline global health score.

^3^ Mean difference (covered - uncovered) calculated using a linear regression model, adjusting for baseline global health score, age, WHO performance status, tumour site, and indication for palliation.

^4^ As in (2) but tumour site variable collapsed into right colon (ascending colon, hepatic flexure, transverse colon, splenic flexure) and left colon/rectum (descending colon, sigmoid, rectosigmoid, rectum (proximal)).

### Stent patency (participants who were adherent to their randomised allocation)

The primary stent patency analysis considered all participants regardless of their adherence to their randomised allocation. This sensitivity analysis restricts the analysis dataset to those participants with successful stent insertions who were adherent to their randomised allocation. This sensitivity analysis has been performed to test the robustness of the primary stent patency analysis.

| **Follow-up period** | **Stent failure** | **covered stent (N=153)** | **uncovered stent (control) (n=161)** | **Unadjusted** | **Adjusted** |
| --- | --- | --- | --- | --- | --- |
|  |  |  |  | **HR (97.5% CI)**  **P-value** | **HR (97.5% CI)^1^**  **P-value** |
| **stent patency up to 6 months** | No | 112 (73.2%) | 133 (82.6%) | 1.58 (0.91, 2.74) | 1.52 (0.87, 2.66) |
|  | Yes | 41 (26.8%) | 28 (17.4%) | P=0.063 | P=0.093 |

^1^Cox proportional hazards model, adjusting for age, tumour site, WHO performance status and indication for palliation. Tumour site has 8 levels in total, which, when adjusted for, caused the model estimates to be unreliable due to small numbers in some categories; therefore, it was collapsed into right colon (ascending colon, hepatic flexure, transverse colon, splenic flexure) and left colon/rectum (descending colon, sigmoid, rectosigmoid, rectum (proximal)). A hazard ratio < 1 favours the covered group.

**QLQ-CR29 quality of life questionnaire**

The data obtained from the QLQ-CR29 questionnaire follows the same analysis method as for the primary outcome – if 3-month data is available then this is used, if 3-month data is missing but 30-day data is available then this is used.

**Body image**

|  | **covered stent** | **uncovered**  **stent**  **(control)** | **Estimate (97.5%CI)** ^1^ | **P-value** |
| --- | --- | --- | --- | --- |
| Number in analysis dataset | 161 | 166 |  |  |
| Number in analysis dataset with a QLQ-CR29 body image score at baseline | 143 | 152 |  |  |
| Number in analysis dataset with a QLQ-CR29 body image score at baseline AND at either 30 days or at month 3 | 97 | 103 |  |  |
| *3 month score available* | *66* | *71* |  |  |
| *30 day score carried forward* | *31* | *32* |  |  |
|  |  |  |  |  |
| Baseline score (mean (SD)) | 85.2 (21.0) | 82.8 (24.7) |  |  |
| 3-month score (mean (SD)) | 75.9 (27.9) | 79.5 (25.2) | -4.52  (-12.48, 3.44) | 0.201 ^2^ |
|  |  |  | -3.63  (-11.72, 4.45) | 0.311 ^3^ |
|  |  |  | -3.35  (-11.23, 4.53) | 0.338 ^4^ |

^1^ Higher mean scores indicate higher functioning. A positive difference favours the covered group. Missing values for body image score at month 3 have been imputed using the corresponding score at 30 days where available.

^2^ Mean difference (covered - uncovered) calculated using a linear regression model adjusted for baseline body image score

^3^ Mean difference (covered - uncovered) calculated using a linear regression model, adjusting for baseline body image score, age, WHO performance status, tumour site, and indication for palliation.

^4^ As in (3) but tumour site variable collapsed into right colon (ascending colon, hepatic flexure, transverse colon, splenic flexure) and left colon/rectum (descending colon, sigmoid, rectosigmoid, rectum (proximal)).

**Anxiety**

|  | **covered stent** | **uncovered**  **stent**  **(control)** | **Estimate (97.5%CI)** ^1^ | **P-value** |
| --- | --- | --- | --- | --- |
| Number in analysis dataset | 161 | 166 |  |  |
| Number in analysis dataset with a QLQ-CR29 anxiety score at baseline | 145 | 151 |  |  |
| Number in analysis dataset with a QLQ-CR29 anxiety score at baseline AND at either 30 days or at month 3 | 99 | 104 |  |  |
| *3 month score available* | *69* | *71* |  |  |
| *30 day score carried forward* | *30* | *33* |  |  |
|  |  |  |  |  |
| Baseline score (mean (SD)) | 48.8 (34.4) | 52.2 (31.4) |  |  |
| 3-month score (mean (SD)) | 55.9 (31.9) | 52.6 (33.7) | 4.63  (-5.02, 14.29) | 0.280 ^2^ |
|  |  |  | 3.38  (-6.68, 13.43) | 0.449 ^3^ |
|  |  |  | 4.41  (-5.46, 14.29) | 0.314 ^4^ |

^1^ Higher mean scores indicate higher functioning. A positive difference favours the covered group. Missing values for anxiety score at month 3 have been imputed using the corresponding score at 30 days where available.

^2^ Mean difference (covered - uncovered) calculated using a linear regression model adjusted for baseline anxiety score

^3^ Mean difference (covered - uncovered) calculated using a linear regression model, adjusting for baseline anxiety score, age, WHO performance status, tumour site, and indication for palliation.

^4^ As in (3) but tumour site variable collapsed into right colon (ascending colon, hepatic flexure, transverse colon, splenic flexure) and left colon/rectum (descending colon, sigmoid, rectosigmoid, rectum (proximal)).

**Weight**

|  | **covered stent** | **uncovered**  **stent**  **(control)** | **Estimate (97.5%CI)** ^1^ | **P-value** |
| --- | --- | --- | --- | --- |
| Number in analysis dataset | 161 | 166 |  |  |
| Number in analysis dataset with a QLQ-CR29 weight score at baseline | 145 | 151 |  |  |
| Number in analysis dataset with a QLQ-CR29 weight score at baseline AND at either 30 days or at month 3 | 100 | 104 |  |  |
| *3 month score available* | *69* | *72* |  |  |
| *30 day score carried forward* | *31* | *32* |  |  |
|  |  |  |  |  |
| Baseline score (mean (SD)) | 73.7 (30.4) | 75.6 (32.6) |  |  |
| 3-month score (mean (SD)) | 74.3 (31.4) | 70.8 (34.7) | 4.50  (-4.69, 13.69) | 0.270 ^2^ |
|  |  |  | 5.11  (-4.54, 14.76) | 0.233 ^3^ |
|  |  |  | 4.03  (-5.35, 13.42) | 0.333 ^4^ |

^1^ Higher mean scores indicate higher functioning. A positive difference favours the covered group. Missing values for weight score at month 3 have been imputed using the corresponding score at 30 days where available.

^2^ Mean difference (covered - uncovered) calculated using a linear regression model adjusted for baseline weight score

^3^ Mean difference (covered - uncovered) calculated using a linear regression model, adjusting for baseline weight score, age, WHO performance status, tumour site, and indication for palliation.

^4^ As in (3) but tumour site variable collapsed into right colon (ascending colon, hepatic flexure, transverse colon, splenic flexure) and left colon/rectum (descending colon, sigmoid, rectosigmoid, rectum (proximal)).

**Sexual Interest (men)**

|  | **covered stent** | **uncovered**  **stent**  **(control)** | **Estimate (97.5%CI)** ^1^ | **P-value** |
| --- | --- | --- | --- | --- |
| Number in analysis dataset | 110 | 101 |  |  |
| Number in analysis dataset with a QLQ-CR29 sexual interest (men) score at baseline | 88 | 75 |  |  |
| Number in analysis dataset with a QLQ-CR29 sexual interest (men)) score at baseline AND at either 30 days or at month 3 | 54 | 48 |  |  |
| *3 month score available* | *35* | *30* |  |  |
| *30 day score carried forward* | *19* | *18* |  |  |
|  |  |  |  |  |
| Baseline score (mean (SD)) | 18.5 (24.8) | 18.7 (32.2) |  |  |
| 3-month score (mean (SD)) | 16.7 (21.2) | 14.6 (20.5) | 2.17  (-6.01, 10.34) | 0.547 ^2^ |
|  |  |  | 0.43  (-8.63, 9.48) | 0.915 ^3^ |
|  |  |  | 0.62  (-8.11, 9.35) | 0.871 ^4^ |

^1^ Higher mean scores indicate higher functioning. A positive difference favours the covered group. Missing values for sexual interest (men) score at month 3 have been imputed using the corresponding score at 30 days where available.

^2^ Mean difference (covered - uncovered) calculated using a linear regression model adjusted for baseline sexual interest (men) score

^3^ Mean difference (covered - uncovered) calculated using a linear regression model, adjusting for baseline sexual interest (men) score, age, WHO performance status, tumour site, and indication for palliation.

^4^ As in (3) but tumour site variable collapsed into right colon (ascending colon, hepatic flexure, transverse colon, splenic flexure) and left colon/rectum (descending colon, sigmoid, rectosigmoid, rectum (proximal)).

**Sexual Interest (women)**

|  | **covered stent** | **uncovered**  **stent**  **(control)** | **Estimate (97.5%CI)** ^1^ | **P-value** |
| --- | --- | --- | --- | --- |
| Number in analysis dataset | 51 | 65 |  |  |
| Number in analysis dataset with a QLQ-CR29 sexual interest (women) score at baseline | 39 | 52 |  |  |
| Number in analysis dataset with a QLQ-CR29 sexual interest (women) score at baseline AND at either 30 days or at month 3 | 29 | 37 |  |  |
| *3 month score available* | *21* | *22* |  |  |
| *30 day score carried forward* | *8* | *15* |  |  |
|  |  |  |  |  |
| Baseline score (mean (SD)) | 2.3 (8.6) | 10.8 (22.3) |  |  |
| 3-month score (mean (SD)) | 6.9 (20.7) | 7.2 (16.0) | 3.47  (-6.21, 13.16) | 0.413 ^2^ |
|  |  |  | 0.31  (-6.76, 7.38) | 0.919 ^3^ |
|  |  |  | 2.32  (-7.93, 12.57) | 0.604 ^4^ |

^1^ Higher mean scores indicate higher functioning. A positive difference favours the covered group. Missing values for sexual interest (women) score at month 3 have been imputed using the corresponding score at 30 days where available.

^2^ Mean difference (covered - uncovered) calculated using a linear regression model adjusted for baseline sexual interest (women) score

^3^ Mean difference (covered - uncovered) calculated using a linear regression model, adjusting for baseline sexual interest (women) score, age, WHO performance status, tumour site, and indication for palliation.

^4^ As in (3) but tumour site variable collapsed into right colon (ascending colon, hepatic flexure, transverse colon, splenic flexure) and left colon/rectum (descending colon, sigmoid, rectosigmoid, rectum (proximal)).

**Urinary Frequency**

|  | **covered stent** | **uncovered**  **stent**  **(control)** | **Estimate (97.5%CI)** ^1^ | **P-value** |
| --- | --- | --- | --- | --- |
| Number in analysis dataset | 161 | 166 |  |  |
| Number in analysis dataset with a QLQ-CR29 urinary frequency score at baseline | 145 | 151 |  |  |
| Number in analysis dataset with a QLQ-CR29 urinary frequency score at baseline AND at either 30 days or at month 3 | 98 | 103 |  |  |
| *3 month score available* | *68* | *71* |  |  |
| *30 day score carried forward* | *30* | *32* |  |  |
|  |  |  |  |  |
| Baseline score (mean (SD)) | 40.0 (27.3) | 40.0 (24.1) |  |  |
| 3-month score (mean (SD)) | 38.9 (26.5) | 40.0 (23.0) | -1.02  (-8.52, 6.48) | 0.759 ^2^ |
|  |  |  | 1.04  (-6.60, 8.67) | 0.759 ^3^ |
|  |  |  | 0.03  (-7.38, 7.45) | 0.992 ^4^ |

^1^ Higher mean scores indicate a higher level of symptoms. A negative difference favours the covered group. Missing values for urinary frequency score at month 3 have been imputed using the corresponding score at 30 days where available.

^2^ Mean difference (covered - uncovered) calculated using a linear regression model adjusted for baseline urinary frequency score

^3^ Mean difference (covered - uncovered) calculated using a linear regression model, adjusting for baseline urinary frequency score, age, WHO performance status, tumour site, and indication for palliation.

^4^ As in (3) but tumour site variable collapsed into right colon (ascending colon, hepatic flexure, transverse colon, splenic flexure) and left colon/rectum (descending colon, sigmoid, rectosigmoid, rectum (proximal)).

**Blood and Mucus in Stools**

|  | **covered stent** | **uncovered stent**  **(control)** | **Estimate (97.5%CI)** ^1^ | **P-value** |
| --- | --- | --- | --- | --- |
| Number in analysis dataset | 161 | 166 |  |  |
| Number in analysis dataset with a QLQ-CR29 blood and mucus score at baseline | 143 | 153 |  |  |
| Number in analysis dataset with a QLQ-CR29 blood and mucus score at baseline AND at either 30 days or at month 3 | 99 | 104 |  |  |
| *3 month score available* | *68* | *72* |  |  |
| *30 day score carried forward* | *31* | *32* |  |  |
|  |  |  |  |  |
| Baseline score (mean (SD)) | 16.5 (26.8) | 14.3 (20.7) |  |  |
| 3-month score (mean (SD)) | 8.2 (14.2) | 10.6 (17.8) | -2.90  (-7.66, 1.87) | 0.171 ^2^ |
|  |  |  | -2.76  (-7.49, 1.97) | 0.189 ^3^ |
|  |  |  | -2.75  (-7.46, 1.96) | 0.189 ^4^ |

^1^ Higher mean scores indicate a higher level of symptoms. A negative difference favours the covered group. Missing values for blood and mucus score at month 3 have been imputed using the corresponding score at 30 days where available.

^2^ Mean difference (covered - uncovered) calculated using a linear regression model adjusted for baseline blood and mucus score

^3^ Mean difference (covered - uncovered) calculated using a linear regression model, adjusting for baseline blood and mucus score, age, WHO performance status, tumour site, and indication for palliation.

^4^ As in (3) but tumour site variable collapsed into right colon (ascending colon, hepatic flexure, transverse colon, splenic flexure) and left colon/rectum (descending colon, sigmoid, rectosigmoid, rectum (proximal)).

**Stool Frequency**

|  | **covered stent** | **uncovered stent**  **(control)** | **Estimate (97.5%CI)** ^1^ | **P-value** |
| --- | --- | --- | --- | --- |
| Number in analysis dataset | 161 | 166 |  |  |
| Number in analysis dataset with a QLQ-CR29 stool frequency score at baseline | 122 | 136 |  |  |
| Number in analysis dataset with a QLQ-CR29 stool frequency score at baseline AND at either 30 days or at month 3 | 79 | 91 |  |  |
| *3 month score available* | *51* | *63* |  |  |
| *30 day score carried forward* | *28* | *28* |  |  |
|  |  |  |  |  |
| Baseline score (mean (SD)) | 23.8 (25.7) | 22.9 (26.9) |  |  |
| 3-month score (mean (SD)) | 25.9 (20.5) | 26.4 (26.8) | -0.75  (-8.53, 7.04) | 0.829 ^2^ |
|  |  |  | -1.80  (-9.83, 6.22) | 0.612 ^3^ |
|  |  |  | -1.50  (-9.32, 6.32) | 0.664 ^4^ |

^1^ Higher mean scores indicate a higher level of symptoms. A negative difference favours the covered group. Missing values for stool frequency score at month 3 have been imputed using the corresponding score at 30 days where available.

^2^ Mean difference (covered - uncovered) calculated using a linear regression model adjusted for baseline stool frequency score

^3^ Mean difference (covered - uncovered) calculated using a linear regression model, adjusting for baseline stool frequency score, age, WHO performance status, tumour site, and indication for palliation.

^4^ As in (3) but tumour site variable collapsed into right colon (ascending colon, hepatic flexure, transverse colon, splenic flexure) and left colon/rectum (descending colon, sigmoid, rectosigmoid, rectum (proximal)).

**Urinary Incontinence**

|  | **covered stent** | **uncovered stent**  **(control)** | **Estimate (97.5%CI)** ^1^ | **P-value** |
| --- | --- | --- | --- | --- |
| Number in analysis dataset | 161 | 166 |  |  |
| Number in analysis dataset with a QLQ-CR29 urinary incontinence score at baseline | 143 | 151 |  |  |
| Number in analysis dataset with a QLQ-CR29 urinary incontinence score at baseline AND at either 30 days or at month 3 | 96 | 103 |  |  |
| *3 month score available* | *66* | *71* |  |  |
| *30 day score carried forward* | *30* | *32* |  |  |
|  |  |  |  |  |
| Baseline score (mean (SD)) | 9.7 (19.3) | 12.0 (20.3) |  |  |
| 3-month score (mean (SD)) | 9.7 (18.0) | 13.3 (22.5) | -2.60  (-8.64, 3.43) | 0.331 ^2^ |
|  |  |  | -1.45  (-7.56, 4.67) | 0.593 ^3^ |
|  |  |  | -2.23  (-8.23, 3.76) | 0.401 ^4^ |

^1^ Higher mean scores indicate a higher level of symptoms. A negative difference favours the covered group. Missing values for urinary incontinence score at month 3 have been imputed using the corresponding score at 30 days where available.

^2^ Mean difference (covered - uncovered) calculated using a linear regression model adjusted for baseline urinary incontinence score

^3^ Mean difference (covered - uncovered) calculated using a linear regression model, adjusting for baseline urinary incontinence score, age, WHO performance status, tumour site, and indication for palliation.

^4^ As in (3) but tumour site variable collapsed into right colon (ascending colon, hepatic flexure, transverse colon, splenic flexure) and left colon/rectum (descending colon, sigmoid, rectosigmoid, rectum (proximal)).

**Dysuria**

|  | **covered stent** | **uncovered stent**  **(control)** | **Estimate (97.5%CI)** ^1^ | **P-value** |
| --- | --- | --- | --- | --- |
| Number in analysis dataset | 161 | 166 |  |  |
| Number in analysis dataset with a QLQ-CR29 dysuria score at baseline | 145 | 150 |  |  |
| Number in analysis dataset with a QLQ-CR29 dysuria score at baseline AND at either 30 days or at month 3 | 97 | 102 |  |  |
| *3 month score available* | *67* | *69* |  |  |
| *30 day score carried forward* | *30* | *33* |  |  |
|  |  |  |  |  |
| Baseline score (mean (SD)) | 3.1 (13.7) | 4.6 (16.3) |  |  |
| 3-month score (mean (SD)) | 3.1 (9.7) | 5.6 (15.6) | -2.28  (-6.45, 1.88) | 0.217 ^2^ |
|  |  |  | -1.84  (-6.07, 2.40) | 0.328 ^3^ |
|  |  |  | -1.94  (-6.10, 2.23) | 0.294 ^4^ |

^1^ Higher mean scores indicate a higher level of symptoms. A negative difference favours the covered group. Missing values for dysuria score at month 3 have been imputed using the corresponding score at 30 days where available.

^2^ Mean difference (covered - uncovered) calculated using a linear regression model adjusted for baseline dysuria score

^3^ Mean difference (covered - uncovered) calculated using a linear regression model, adjusting for baseline dysuria score, age, WHO performance status, tumour site, and indication for palliation.

^4^ As in (3) but tumour site variable collapsed into right colon (ascending colon, hepatic flexure, transverse colon, splenic flexure) and left colon/rectum (descending colon, sigmoid, rectosigmoid, rectum (proximal)).

**Abdominal Pain**

|  | **covered stent** | **uncovered stent**  **(control)** | **Estimate (97.5%CI)** ^1^ | **P-value** |
| --- | --- | --- | --- | --- |
| Number in analysis dataset | 161 | 166 |  |  |
| Number in analysis dataset with a QLQ-CR29 abdominal pain score at baseline | 147 | 153 |  |  |
| Number in analysis dataset with a QLQ-CR29 abdominal pain score at baseline AND at either 30 days or at month 3 | 100 | 104 |  |  |
| *3 month score available* | *69* | *71* |  |  |
| *30 day score carried forward* | *31* | *33* |  |  |
|  |  |  |  |  |
| Baseline score (mean (SD)) | 42.0 (37.5) | 36.9 (36.5) |  |  |
| 3-month score (mean (SD)) | 22.3 (29.2) | 23.4 (30.1) | -1.89  (-11.12, 7.35) | 0.645 ^2^ |
|  |  |  | -2.28  (-11.88, 7.32) | 0.593 ^3^ |
|  |  |  | -2.08  (-11.52, 7.37) | 0.620 ^4^ |

^1^ Higher mean scores indicate a higher level of symptoms. A negative difference favours the covered group. Missing values for abdominal pain score at month 3 have been imputed using the corresponding score at 30 days where available.

^2^ Mean difference (covered - uncovered) calculated using a linear regression model adjusted for baseline abdominal pain score

^3^ Mean difference (covered - uncovered) calculated using a linear regression model, adjusting for baseline abdominal pain score, age, WHO performance status, tumour site, and indication for palliation.

^4^ As in (3) but tumour site variable collapsed into right colon (ascending colon, hepatic flexure, transverse colon, splenic flexure) and left colon/rectum (descending colon, sigmoid, rectosigmoid, rectum (proximal)).

**Buttock Pain**

|  | **covered stent** | **uncovered stent**  **(control)** | **Estimate (97.5%CI)** ^1^ | **P-value** |
| --- | --- | --- | --- | --- |
| Number in analysis dataset | 161 | 166 |  |  |
| Number in analysis dataset with a QLQ-CR29 buttock pain score at baseline | 146 | 152 |  |  |
| Number in analysis dataset with a QLQ-CR29 buttock pain score at baseline AND at either 30 days or at month 3 | 99 | 104 |  |  |
| *3 month score available* | *69* | *72* |  |  |
| *30 day score carried forward* | *30* | *32* |  |  |
|  |  |  |  |  |
| Baseline score (mean (SD)) | 15.5 (25.3) | 14.4 (24.5) |  |  |
| 3-month score (mean (SD)) | 16.2 (24.9) | 12.5 (25.5) | 3.32  (-4.29, 10.93) | 0.326 ^2^ |
|  |  |  | 2.05  (-5.81, 9.90) | 0.556 ^3^ |
|  |  |  | 2.88  (-4.94, 10.69) | 0.407 ^4^ |

^1^ Higher mean scores indicate a higher level of symptoms. A negative difference favours the covered group. Missing values for buttock pain score at month 3 have been imputed using the corresponding score at 30 days where available.

^2^ Mean difference (covered - uncovered) calculated using a linear regression model adjusted for baseline buttock pain score

^3^ Mean difference (covered - uncovered) calculated using a linear regression model, adjusting for baseline buttock pain score, age, WHO performance status, tumour site, and indication for palliation.

^4^ As in (3) but tumour site variable collapsed into right colon (ascending colon, hepatic flexure, transverse colon, splenic flexure) and left colon/rectum (descending colon, sigmoid, rectosigmoid, rectum (proximal)).

**Bloating**

|  | **covered stent** | **uncovered stent**  **(control)** | **Estimate (97.5%CI)** ^1^ | **P-value** |
| --- | --- | --- | --- | --- |
| Number in analysis dataset | 161 | 166 |  |  |
| Number in analysis dataset with a QLQ-CR29 bloating score at baseline | 144 | 153 |  |  |
| Number in analysis dataset with a QLQ-CR29 bloating score at baseline AND at either 30 days or at month 3 | 99 | 104 |  |  |
| *3 month score available* | *68* | *72* |  |  |
| *30 day score carried forward* | *31* | *32* |  |  |
|  |  |  |  |  |
| Baseline score (mean (SD)) | 47.1 (36.9) | 47.8 (39.9) |  |  |
| 3-month score (mean (SD)) | 28.6 (29.7) | 31.1 (32.3) | -2.36  (-11.96, 7.25) | 0.581 ^2^ |
|  |  |  | -2.23  (-12.04, 7.58) | 0.608 ^3^ |
|  |  |  | -2.23  (-12.02, 7.56) | 0.608 ^4^ |

^1^ Higher mean scores indicate a higher level of symptoms. A negative difference favours the covered group. Missing values for bloating score at month 3 have been imputed using the corresponding score at 30 days where available.

^2^ Mean difference (covered - uncovered) calculated using a linear regression model adjusted for baseline bloating score

^3^ Mean difference (covered - uncovered) calculated using a linear regression model, adjusting for baseline bloating score, age, WHO performance status, tumour site, and indication for palliation.

^4^ As in (3) but tumour site variable collapsed into right colon (ascending colon, hepatic flexure, transverse colon, splenic flexure) and left colon/rectum (descending colon, sigmoid, rectosigmoid, rectum (proximal)).

**Dry Mouth**

|  | **covered stent** | **uncovered stent**  **(control)** | **Estimate (97.5%CI)** ^1^ | **P-value** |
| --- | --- | --- | --- | --- |
| Number in analysis dataset | 161 | 166 |  |  |
| Number in analysis dataset with a QLQ-CR29 dry mouth score at baseline | 145 | 151 |  |  |
| Number in analysis dataset with a QLQ-CR29 dry mouth score at baseline AND at either 30 days or at month 3 | 99 | 104 |  |  |
| *3 month score available* | *69* | *72* |  |  |
| *30 day score carried forward* | *30* | *32* |  |  |
|  |  |  |  |  |
| Baseline score (mean (SD)) | 42.1 (38.0) | 40.1 (36.1) |  |  |
| 3-month score (mean (SD)) | 30.3 (30.2) | 32.7 (30.8) | -2.93  (-12.11, 6.25) | 0.472 ^2^ |
|  |  |  | -3.08  (-12.46, 6.31) | 0.459 ^3^ |
|  |  |  | -3.07  (-12.32, 6.18) | 0.454 ^4^ |

^1^ Higher mean scores indicate a higher level of symptoms. A negative difference favours the covered group. Missing values for dry mouth score at month 3 have been imputed using the corresponding score at 30 days where available.

^2^ Mean difference (covered - uncovered) calculated using a linear regression model adjusted for baseline dry mouth score

^3^ Mean difference (covered - uncovered) calculated using a linear regression model, adjusting for baseline dry mouth score, age, WHO performance status, tumour site, and indication for palliation.

^4^ As in (3) but tumour site variable collapsed into right colon (ascending colon, hepatic flexure, transverse colon, splenic flexure) and left colon/rectum (descending colon, sigmoid, rectosigmoid, rectum (proximal)).

**Hair Loss**

|  | **covered stent** | **uncovered stent**  **(control)** | **Estimate (97.5%CI)** ^1^ | **P-value** |
| --- | --- | --- | --- | --- |
| Number in analysis dataset | 161 | 166 |  |  |
| Number in analysis dataset with a QLQ-CR29 hair loss score at baseline | 143 | 147 |  |  |
| Number in analysis dataset with a QLQ-CR29 hair loss score at baseline AND at either 30 days or at month 3 | 99 | 100 |  |  |
| *3 month score available* | *66* | *70* |  |  |
| *30 day score carried forward* | *33* | *30* |  |  |
|  |  |  |  |  |
| Baseline score (mean (SD)) | 5.1 (16.1) | 1.7 (7.3) |  |  |
| 3-month score (mean (SD)) | 13.5 (26.9) | 9.0 (17.6) | 3.90  (-3.43, 11.24) | 0.231 ^2^ |
|  |  |  | 2.29  (-4.84, 9.42) | 0.468 ^3^ |
|  |  |  | 2.63  (-4.38, 9.64) | 0.397 ^4^ |

^1^ Higher mean scores indicate a higher level of symptoms. A negative difference favours the covered group. Missing values for hair loss score at month 3 have been imputed using the corresponding score at 30 days where available.

^2^ Mean difference (covered - uncovered) calculated using a linear regression model adjusted for baseline hair loss score

^3^ Mean difference (covered - uncovered) calculated using a linear regression model, adjusting for baseline hair loss score, age, WHO performance status, tumour site, and indication for palliation.

^4^ As in (3) but tumour site variable collapsed into right colon (ascending colon, hepatic flexure, transverse colon, splenic flexure) and left colon/rectum (descending colon, sigmoid, rectosigmoid, rectum (proximal)).

**Taste**

|  | **covered stent** | **uncovered stent**  **(control)** | **Estimate (97.5%CI)** ^1^ | **P-value** |
| --- | --- | --- | --- | --- |
| Number in analysis dataset | 161 | 166 |  |  |
| Number in analysis dataset with a QLQ-CR29 taste score at baseline | 143 | 153 |  |  |
| Number in analysis dataset with a QLQ-CR29 taste score at baseline AND at either 30 days or at month 3 | 100 | 104 |  |  |
| *3 month score available* | *69* | *72* |  |  |
| *30 day score carried forward* | *31* | *32* |  |  |
|  |  |  |  |  |
| Baseline score (mean (SD)) | 16.3 (28.6) | 12.8 (24.7) |  |  |
| 3-month score (mean (SD)) | 26.0 (32.7) | 23.1 (28.7) | 1.89  (-7.54, 11.32) | 0.651 ^2^ |
|  |  |  | 2.13  (-7.57, 11.82) | 0.621 ^3^ |
|  |  |  | 1.89  (-7.55, 11.33) | 0.652 ^4^ |

^1^ Higher mean scores indicate a higher level of symptoms. A negative difference favours the covered group. Missing values for taste score at month 3 have been imputed using the corresponding score at 30 days where available.

^2^ Mean difference (covered - uncovered) calculated using a linear regression model adjusted for baseline taste score

^3^ Mean difference (covered - uncovered) calculated using a linear regression model, adjusting for baseline taste score, age, WHO performance status, tumour site, and indication for palliation.

^4^ As in (3) but tumour site variable collapsed into right colon (ascending colon, hepatic flexure, transverse colon, splenic flexure) and left colon/rectum (descending colon, sigmoid, rectosigmoid, rectum (proximal)).

**Flatulence**

|  | **covered stent** | **uncovered stent**  **(control)** | **Estimate (97.5%CI)** ^1^ | **P-value** |
| --- | --- | --- | --- | --- |
| Number in analysis dataset | 161 | 166 |  |  |
| Number in analysis dataset with a QLQ-CR29 flatulence score at baseline | 123 | 135 |  |  |
| Number in analysis dataset with a QLQ-CR29 flatulence score at baseline AND at either 30 days or at month 3 | 79 | 90 |  |  |
| *3 month score available* | *52* | *63* |  |  |
| *30 day score carried forward* | *27* | *27* |  |  |
|  |  |  |  |  |
| Baseline score (mean (SD)) | 30.4 (32.6) | 37.4 (32.7) |  |  |
| 3-month score (mean (SD)) | 34.2 (31.6) | 36.3 (31.9) | 0.30  (-10.13, 10.74) | 0.947 ^2^ |
|  |  |  | 0.50  (-10.34, 11.35) | 0.917 ^3^ |
|  |  |  | 0.11  (-10.39, 10.62) | 0.981 ^4^ |

^1^ Higher mean scores indicate a higher level of symptoms. A negative difference favours the covered group. Missing values for flatulence score at month 3 have been imputed using the corresponding score at 30 days where available.

^2^ Mean difference (covered - uncovered) calculated using a linear regression model adjusted for baseline flatulence score

^3^ Mean difference (covered - uncovered) calculated using a linear regression model, adjusting for baseline flatulence score, age, WHO performance status, tumour site, and indication for palliation.

^4^ As in (3) but tumour site variable collapsed into right colon (ascending colon, hepatic flexure, transverse colon, splenic flexure) and left colon/rectum (descending colon, sigmoid, rectosigmoid, rectum (proximal)).

**Faecal Incontinence**

|  | **covered stent** | **uncovered stent**  **(control)** | **Estimate (97.5%CI)** ^1^ | **P-value** |
| --- | --- | --- | --- | --- |
| Number in analysis dataset | 161 | 166 |  |  |
| Number in analysis dataset with a QLQ-CR29 faecal incontinence score at baseline | 122 | 136 |  |  |
| Number in analysis dataset with a QLQ-CR29 faecal incontinence score at baseline AND at either 30 days or at month 3 | 78 | 92 |  |  |
| *3 month score available* | *50* | *62* |  |  |
| *30 day score carried forward* | *28* | *30* |  |  |
|  |  |  |  |  |
| Baseline score (mean (SD)) | 19.2 (30.2) | 17.0 (27.3) |  |  |
| 3-month score (mean (SD)) | 16.7 (25.1) | 15.6 (25.9) | 0.49  (-8.00, 8.99) | 0.896 ^2^ |
|  |  |  | 0.74  (-7.58, 9.06) | 0.841 ^3^ |
|  |  |  | 1.13  (-7.23, 9.49) | 0.759 ^4^ |

^1^ Higher mean scores indicate a higher level of symptoms. A negative difference favours the covered group. Missing values for faecal incontinence score at month 3 have been imputed using the corresponding score at 30 days where available.

^2^ Mean difference (covered - uncovered) calculated using a linear regression model adjusted for baseline faecal incontinence score

^3^ Mean difference (covered - uncovered) calculated using a linear regression model, adjusting for baseline faecal incontinence score, age, WHO performance status, tumour site, and indication for palliation.

^4^ As in (3) but tumour site variable collapsed into right colon (ascending colon, hepatic flexure, transverse colon, splenic flexure) and left colon/rectum (descending colon, sigmoid, rectosigmoid, rectum (proximal)).

**Sore Skin**

|  | **covered stent** | **uncovered stent**  **(control)** | **Estimate (97.5%CI)** ^1^ | **P-value** |
| --- | --- | --- | --- | --- |
| Number in analysis dataset | 161 | 166 |  |  |
| Number in analysis dataset with a QLQ-CR29 sore skin score at baseline | 122 | 136 |  |  |
| Number in analysis dataset with a QLQ-CR29 sore skin score at baseline AND at either 30 days or at month 3 | 78 | 92 |  |  |
| *3 month score available* | *52* | *63* |  |  |
| *30 day score carried forward* | *26* | *29* |  |  |
|  |  |  |  |  |
| Baseline score (mean (SD)) | 6.8 (17.3) | 10.9 (22.7) |  |  |
| 3-month score (mean (SD)) | 16.2 (25.1) | 14.9 (25.4) | 2.27  (-6.44, 10.98) | 0.556 ^2^ |
|  |  |  | 1.49  (-7.30, 10.28) | 0.701 ^3^ |
|  |  |  | 0.87  (-7.80, 9.54) | 0.821 ^4^ |

^1^ Higher mean scores indicate a higher level of symptoms. A negative difference favours the covered group. Missing values for sore skin score at month 3 have been imputed using the corresponding score at 30 days where available.

^2^ Mean difference (covered - uncovered) calculated using a linear regression model adjusted for baseline sore skin score

^3^ Mean difference (covered - uncovered) calculated using a linear regression model, adjusting for baseline sore skin score, age, WHO performance status, tumour site, and indication for palliation.

^4^ As in (3) but tumour site variable collapsed into right colon (ascending colon, hepatic flexure, transverse colon, splenic flexure) and left colon/rectum (descending colon, sigmoid, rectosigmoid, rectum (proximal)).

**Embarrassment**

|  | **covered stent** | **uncovered stent**  **(control)** | **Estimate (97.5%CI)** ^1^ | **P-value** |
| --- | --- | --- | --- | --- |
| Number in analysis dataset | 161 | 166 |  |  |
| Number in analysis dataset with a QLQ-CR29 embarrassment score at baseline | 120 | 133 |  |  |
| Number in analysis dataset with a QLQ-CR29 embarrassment score at baseline AND at either 30 days or at month 3 | 78 | 90 |  |  |
| *3 month score available* | *50* | *62* |  |  |
| *30 day score carried forward* | *28* | *28* |  |  |
|  |  |  |  |  |
| Baseline score (mean (SD)) | 17.5 (28.8) | 18.1 (29.6) |  |  |
| 3-month score (mean (SD)) | 18.4 (30.2) | 17.8 (27.5) | 0.74  (-9.08, 10.56) | 0.865 ^2^ |
|  |  |  | -0.02  (-10.01, 9.96) | 0.996 ^3^ |
|  |  |  | 0.31  (-9.49, 10.10) | 0.943 ^4^ |

^1^ Higher mean scores indicate a higher level of symptoms. A negative difference favours the covered group. Missing values for embarrassment score at month 3 have been imputed using the corresponding score at 30 days where available.

^2^ Mean difference (covered - uncovered) calculated using a linear regression model adjusted for baseline embarrassment score

^3^ Mean difference (covered - uncovered) calculated using a linear regression model, adjusting for baseline embarrassment score, age, WHO performance status, tumour site, and indication for palliation.

^4^ As in (3) but tumour site variable collapsed into right colon (ascending colon, hepatic flexure, transverse colon, splenic flexure) and left colon/rectum (descending colon, sigmoid, rectosigmoid, rectum (proximal)).

**Stoma Care Problems**

|  | **covered stent** | **uncovered stent**  **(control)** | **Estimate (97.5%CI)** ^1^ | **P-value** |
| --- | --- | --- | --- | --- |
| Number in analysis dataset | 161 | 166 |  |  |
| Number in analysis dataset with a QLQ-CR29 stoma care score at baseline | 1 | 1 |  |  |
| Number in analysis dataset with a QLQ-CR29 stoma care score at baseline AND at either 30 days or at month 3 | 0 | 1 |  |  |
| *3 month score available* | *0* | *0* |  |  |
| *30 day score carried forward* | *0* | *1* |  |  |
|  |  |  |  |  |
| Baseline score (mean (SD)) | - | - |  |  |
| 3-month score (mean (SD)) | - | - | - | - |
|  |  |  | - | - |
|  |  |  | - | - |

^1^ Higher mean scores indicate a higher level of symptoms. A negative difference favours the covered group. Missing values for stoma care score at month 3 have been imputed using the corresponding score at 30 days where available. The stoma care score cannot be analysed due to only one observation available.

**Impotence (men)**

|  | **covered stent** | **uncovered stent**  **(control)** | **Estimate (97.5%CI)** ^1^ | **P-value** |
| --- | --- | --- | --- | --- |
| Number in analysis dataset | 110 | 101 |  |  |
| Number in analysis dataset with a QLQ-CR29 impotence (men) score at baseline | 80 | 68 |  |  |
| Number in analysis dataset with a QLQ-CR29 impotence (men) score at baseline AND at either 30 days or at month 3 | 48 | 37 |  |  |
| *3 month score available* | *28* | *25* |  |  |
| *30 day score carried forward* | *20* | *12* |  |  |
|  |  |  |  |  |
| Baseline score (mean (SD)) | 25.7 (37.2) | 43.2 (42.9) |  |  |
| 3-month score (mean (SD)) | 37.5 (42.2) | 37.8 (40.2) | 6.77  (-12.82, 26.36) | 0.432 ^2^ |
|  |  |  | 6.65  (-13.68, 26.98) | 0.456 ^3^ |
|  |  |  | 8.24  (-11.40, 27.87) | 0.341 ^4^ |

^1^ Higher mean scores indicate a higher level of symptoms. A negative difference favours the covered group. Missing values for impotence (men) score at month 3 have been imputed using the corresponding score at 30 days where available.

^2^ Mean difference (covered - uncovered) calculated using a linear regression model adjusted for baseline impotence (men) score

^3^ Mean difference (covered - uncovered) calculated using a linear regression model, adjusting for baseline impotence (men) score, age, WHO performance status, tumour site, and indication for palliation.

^4^ As in (3) but tumour site variable collapsed into right colon (ascending colon, hepatic flexure, transverse colon, splenic flexure) and left colon/rectum (descending colon, sigmoid, rectosigmoid, rectum (proximal)).

**Dyspareunia (women)**

|  | **covered stent** | **uncovered stent**  **(control)** | **Estimate (97.5%CI)** ^1^ | **P-value** |
| --- | --- | --- | --- | --- |
| Number in analysis dataset | 51 | 65 |  |  |
| Number in analysis dataset with a QLQ-CR29 dyspareunia (women) score at baseline | 33 | 40 |  |  |
| Number in analysis dataset with a QLQ-CR29 dyspareunia (women) score at baseline AND at either 30 days or at month 3 | 17 | 22 |  |  |
| *3 month score available* | *10* | *10* |  |  |
| *30 day score carried forward* | *7* | *12* |  |  |
|  |  |  |  |  |
| Baseline score (mean (SD)) | 2.0 (8.1) | 12.1 (30.1) |  |  |
| 3-month score (mean (SD)) | 2.0 (8.1) | 12.1 (21.9) | -6.76  (-18.93, 5.41) | 0.202 ^2^ |
|  |  |  | -7.83  (-19.80, 4.15) | 0.131 ^3^ |
|  |  |  | -8.49  (-18.70, 1.72) | 0.059 ^4^ |

^1^ Higher mean scores indicate a higher level of symptoms. A negative difference favours the covered group. Missing values for dyspareunia (women) score at month 3 have been imputed using the corresponding score at 30 days where available.

^2^ Mean difference (covered - uncovered) calculated using a linear regression model adjusted for baseline dyspareunia (women) score

^3^ Mean difference (covered - uncovered) calculated using a linear regression model, adjusting for baseline dyspareunia (women) score, age, WHO performance status, tumour site, and indication for palliation.

^4^ As in (3) but tumour site variable collapsed into right colon (ascending colon, hepatic flexure, transverse colon, splenic flexure) and left colon/rectum (descending colon, sigmoid, rectosigmoid, rectum (proximal)).

**Supplementary Appendixes**

**Collaborators**

**Chief Investigator:** James Hill (Manchester University NHS Foundation Trust)

**Trial management committee and writing committee (alphabetical by surname):** Nicola Fearnhead (Cambridge University Hospitals NHS Foundation Trust), Richard Gray (Clinical Trial Service Unit, University of Oxford), Kelly Handley (Birmingham Clinical Trials Unit, University of Birmingham), James Hill (Central Manchester University Hospitals NHS Foundation Trust), Manjinder Kaur (Birmingham Clinical Trials Unit, University of Birmingham), Clive Kay (Kings College Hospital NHS Foundation Trust), Hans-Ulrich Laasch (The Christie NHS Foundation Trust, Manchester), Andrew Lowe (Taunton and Somerset NHS Foundation Trust), Laura Magill (Birmingham Clinical Trials Unit, University of Birmingham), Dion Morton (University Hospitals Birmingham NHS Foundation Trust), Ruben Mujica-Mota (Leeds Institute of Health Sciences, University of Leeds), Andy Palmer (Birmingham Clinical Trials Unit, University of Birmingham), Anne Pullyblank (North Bristol NHS Trust), Yongzhong Sun (Birmingham Clinical Trials Unit, University of Birmingham), Suresh Vasan Venkatachalapathy (Nottingham University Hospitals NHS Trust).

**Independent patient advocate:** Pete Wheatstone

**University of Birmingham Clinical Trials Unit:** Yasmin Ali, Altus Chan, Manjinder Kaur, Suzanne Locker, Laura Magill, Andy Palmer (Trials Management), Kelly Handley, Yongzhong Sun (Statistical analysis), Paul Riley (Programming)

**Data Monitoring and Ethics Committee:** Gordon Carlson (Chair), Louise Hiller, Stuart Taylor.

**Trial Steering Committee:** Sarah Barry, Philip Bell, Steve Halligan, Nigel Scott (Chair).

**Participating centres and investigators (*principal investigator at each centre) (alphabetical by trust and then surname):** Aintree University Hospitals NHS Foundation Trust; Suhail Ahmed*, James Arthur, Carol Brooks, Jane L Hughes, Tanya Ingram, Michelle Linforth, Sophie Marsh, Rizwan Saleem, Simone Slawik, Sarah Stevenson, Lilian Wajero, Aneurin Bevan University Health Board; Nicholas Cross*, Amanda Dell, Mandy Edwards, Angela Hall, Helen Hamilton, Nancy Hawkins, Heidi Lawson, Mark Robinson, Michelle Tayler, Rebecca Wallace, Sarah Wheatman*, Joanna Wilson, Lindianne Aitken, Rhodri Codd, Joseph Hamill*, Nancy Hawkins, Georgia Mallison, Steve McKain, Heeam Nassa, Claire Louise Price, Mark Robinson, Brian Stephenson, Keshav Swarnkar, Claire Triscott, Elaine Wall, Rebecca Wallace, Gethin Williams, Cerian Williams, Barking, Havering and Redbridge University Hospitals NHS Trust; Rommel Butawan, Joseph Huang, Sam King, Tina Mills-Baldock, Purushothaman Premchand*, Alison Ray, Blackpool Teaching Hospitals NHS Foundation Trust; Amy Barnett, Alexander Blackmore*, Oliver Brennan, Melaine Caswell, Greta Van Doyvenvoorde, James Glen, Shamina Hankinson, Mark Hendrickse, Peter Isaacs, Ilianna Mamali, Senthil Murugesan, Marina Oprea, Chris Pemberton, Ella Riedel, Arunan Sivapataham, Wei Fen Tay, Lauren Thornborough, Rachel Wheeldon, Conor Wilkinson, Bolton NHS Foundation Trust; Julie Chadwick, Shirley Cocks, Gemma Faulkner, Robert Hull, Marta Martinez Iglesias, James Lay, Ha Phuong Do Le, James Pollard*, Shenraga Kumar Rajamanickam, Rubeena Razzaq, Michaela Sutherland, Bradford Teaching Hospitals NHS Foundation Trust; Aphan Abdulholim, Conrad Beckett*, Wendy Cardozo, Carol Firth, Naeem Jagirdar, Wendy Jepson, Sarah Jowett, Amjad Mohammed, Sulleman Moreea, Nicolas Rabb, Jonathan Robinson, Sophie Stephenson, Sarah Tinker, Cambridge University Hospitals NHS Foundation Trust; Ashlea Bucke, Ewen Cameron, Nicholas Carroll, Gareth Corbett, Nicola Fearnhead*, Nigel Hall, Alisa Liddle, Ines Modolell, Jonathan Morton, Aileen Nacorda, Sophie Newton, Beverley Nobbs, Debbie Read, Rebekka Troller, James Wheeler, Lucy Worboys, Central Manchester University Hospitals NHS Foundation Trust; Megan Brickhill, Chris Craig, Finlay Curran, David Donnelly, Mona Fareh, Bethanie Garside, Glaxy Gray, Richard Hammonds, Babra Hanif, James Hill*, Benjamin Hornung, Anu John, Maya John, Stephen Lee, Jesha Mathews, Pavenjit Nandhra, Mohammed Nazir, Jessica Nichols, Sarah O'Shea, Alice Panes, Laura Perry, Angelique Quistin, Rojy Santosh, Nicholas Stylianides, Jennifer Trezise, Denielle Wilcock, Countess of Chester Hospital NHS Foundation Trust; Gian Abbott, Paul Evans, Claire Gabriel, Jenny Grounds, Nichola Kearsley, Roy Mahapatra, Collette Markzu, Emmeline Martin, Laura Parry, Sandra Powell, Kunal Rajput, Dale Vimalachandran*, Andrea Young, East Cheshire NHS Trust; Helen Boros, Lisa Hardstaff, Philippa Hill, Maureen Holland, Debra Jowle, Konrad Koss*, Barbara Townley, Lesley Wilknson, Hampshire Hospitals NHS Foundation Trust; Hayley Cousins, Barbara King, John Ramage*, Lancashire Teaching Hospitals NHS Foundation Trust; Amanda Alty, Paul Barrow, Alan Beveridge, Arnab Bhowmick, Alistair Craig, Terri-Louise Cromie, Tarek Hany, Alka Jadav, Janet Mills, Peter Mitchell*, Ed Parkin, Ioannis Peristerakis, Sandra Sowden, Robert Stockwell, Gagandeep Thind, Louis Turrel, Mark Verlander, Ailsa Watt, Deborah Weavers, Alexandra Williams, Leeds Teaching Hospitals NHS Trust; Miranda Baum, Simon Everett, Vinod Hegade, Matthew Huggett, Susan Kelly, Rebecca King, Lucy Marshall, Catherine Moriarty, Bharat Paranandi, Mark Priestley, Rick Saunders*, Holly Speight, Louise White, Manchester University NHS Foundation Trust; Haidar Alwan-Walker, Helen Ashby, Linda Bailey, Wal Baraza, Molly Bennett, Angela Chrisopoulou, Sarah Duff, Laura Hancock, James Hill*, Zoe Holliday, Javaid Iqbal*, Venkata Lekharaju, Fiona McCartin, Gorei Mccavil, Stephen Metcalfe, Heena Mistry, Lindsay Piper, Aswatha Ramesh, Velauthan Rudralingam, Abhiram Sharma, Kathryn Slevin, Karen Telford, Debbie West, Kate Whitehead, Newcastle upon Tyne Hospitals NHS Foundation Trust; Peter Coyne, James Graham, Stephanie Grieveson, Ben Griffiths, Lorna Ingoe, Sam McDonald, Victoria Murtha, Adam Scadeng, Julia Scott, Elaine Stephenson, Vithusa Varnakillasingam, Nelson Wong, NHS Highland; Avril Donaldson, Kathleen Macleod, Andrew Macleod, Joanna Matheson, Raymond Oliphant, Alastair Todd, Michael Walker, Angus Watson*, NHS Lothian; Angie Balfour, Domenyk Brown, John Brush, Stephen Glancy, Sarah Goodbrand, Marion MacRury, Chinnappa Reddy, Doug Speake, Geoff Wogan, North Bristol NHS Trust; Sarah Bevins, Kirstie Bradburn, Caroline Burt, Neil Collin, Graham Collin, Laura Fox, Robert Healey, Mitchell Hopes, Shinu Jackson, Alice Jarvie, Regina Kageni, Suriya Kirkpatrick, Sam Loud, Eric Loveday, Ann Lyons, Kathryn McCarthy, Tom McGirr, Angus McNair, Peter Mezes, Emily Perry, Anne Pullyblank*, Sosamma Robin, Maricruz Santamaria, Alice Smith, Andrew Smith, Louise Solomon, Haytham Sumrien, Isileli Tonga*, Katherine Way, David Westwood, Nottingham University Hospitals NHS Trust; Guruprasad P Aithal, Andrew Baxter, Suzanne Henry, Martin James, Amardeep Khanna, Jodie Newham, Sian Kelly Parkes, Stephen Ryder, Ioannis Varmpompitis, Suresh Vasan Venkatachalapathy*, Samantha Warburton, Royal Cornwall Hospitals NHS Trust; Sarah Askew, Kerry Atkinson, Madalina Chifu, Candy Coombe, Sophia Eloi, Clare Ferris, Elizabeth Firth, Caroline Goddard, Anne Griffiths, John Hancock*, Angela Irving, Kirsty Maclean, John Madine, Corrine Penhaligon, Catherine Pentescost, Kirsty Prout, Rebecca Rogers, Rebecca Sargent, Anita Steele, Felicity Verma, Sheffield Teaching Hospitals NHS Foundation Trust; Michael Agyemang, Sarah Bird, Steven Brown*, Holly Caborn, Joyce Fofie, James Hampton, Faith Kibutu, Fred Lee, Cecilia Mason, Angeline Mbuyisa, Helen Newell, Somerset NHS Foundation Trust; Viktoria Cripps, Thomas Edwards, Nicky Forsyth, Louise Hunt, Andrew Lowe, Paul Mackey, Rudi Matull*, Alison Moss, Corinne Pawley, Tamlyn Russell, Maria Salter, Charmaine Shovelton, Edward Smyth, South Eastern Health and Social Care Trust; Angela Berry, Nicola Broome, Grant Caddy*, John Eccles, Jennifer Foreman, Tony Tham, Alex Usher-Rea, Andrew Wray, Gail Young, The Christie NHS Foundation Trust; Harry Bond, Theresa Taylor Emberton, Hans-Ulrich Laasch*, Sue Lane (Nee Fenton), Damian Mullan, Lyne Robertson, Maria RoyoGamara, The Royal Wolverhampton NHS Trust; Marie Green, Brian McKaig, Shyam Menon*, Rajinder Nayyar, Julie Roberts, Helen Steed, Andrew Veitch, University Hospitals Birmingham NHS Foundation Trust; Sarah Addison, Shazad Ashraf, Simon Bach, Anil Bagul, Elizabeth Bailey, Andrew Beggs, Colm Forde*, Sharon Garner, Manijeh Ghods, Andrew McDarby, Claire McNeill, Dion Morton, Dimitri Nepogodiev, Arvind Pallan, Tom Pinkney, Jonathan Richardson, Nigel Suggett, Sharan Wadhwani, Stephan Ward, Arlo Whithouse, Deborah Wright, University Hospitals of Derby and Burton NHS; Hasan Al Chalabi, Ashish Bhalla, Jo Chmiel, Julie Edmonds, Jodie Fitzgerald, Nicole Isitt, Jonathan Lund, Nicole Mckee, Joely Morgan, Elizabeth Nadin, Ellie Piggott, Rajeev Singh*, Katherine Smith, William Speake, Peter Thurley, Samson Tou, Christ Worth, University Hospitals of Leicester NHS Trust; Jill Cooke, Rachel Plummer, Baljit Singh*, Ratan Verma, University Hospitals of North Midlands NHS Trust; Ndkeita Barnett, Adrian Butler, Susan Gallagher, Amanda Hall, Kar Wai Lau, Mia Marsden, Michael Martin, Katrina Parkinson, Rochelle Rhodes, Alison Tilley, York and Scarborough Teaching Hospitals NHS Foundation Trust; Laura Barman, Kerry Elliot, Janine Mallinson, Tania Neale, Ian Renwick*, Jacqui Smith, Alison Turnbull.
